# Alveolar regeneration through a Krt8+ transitional stem cell state that persists in human lung fibrosis

**DOI:** 10.1038/s41467-020-17358-3

**Published:** 2020-07-16

**Authors:** Maximilian Strunz, Lukas M. Simon, Meshal Ansari, Jaymin J. Kathiriya, Ilias Angelidis, Christoph H. Mayr, George Tsidiridis, Marius Lange, Laura F. Mattner, Min Yee, Paulina Ogar, Arunima Sengupta, Igor Kukhtevich, Robert Schneider, Zhongming Zhao, Carola Voss, Tobias Stoeger, Jens H. L. Neumann, Anne Hilgendorff, Jürgen Behr, Michael O’Reilly, Mareike Lehmann, Gerald Burgstaller, Melanie Königshoff, Harold A. Chapman, Fabian J. Theis, Herbert B. Schiller

**Affiliations:** 1 Institute of Lung Biology and Disease and Comprehensive Pneumology Center with the CPC-M bioArchive, Helmholtz Zentrum Muenchen, Member of the German Center for Lung Research (DZL), Munich, Germany; 2 Institute of Computational Biology, Helmholtz Zentrum München, Munich, Germany; 3 Center for Precision Health, School of Biomedical Informatics, University of Texas Health Science Center, Houston, TX USA; 4 Biomedical Center, University of California San Francisco, San Francisco, CA USA; 5 Department of Mathematics, Technische Universität München, Munich, Germany; 6 Department of Pediatrics, University of Rochester, Rochester, NY USA; 7 Institute of Functional Epigenetics, Helmholtz Zentrum München, Munich, Germany; 8 Institute of Pathology, Ludwig Maximilians University Hospital Munich, Munich, Germany; 9 Member of the German Center for Lung Research (DZL), Center for Comprehensive Developmental Care (CDeCLMU), Department of Neonatology, Perinatal Center Grosshadern, Hospital of the Ludwig-Maximilians University (LMU), Munich, Germany; 10 Member of the German Center for Lung Research (DZL), Department of Internal Medicine V, Ludwig Maximilians University Hospital (LMU) Munich, Munich, Germany; 11 Asklepios Fachkliniken in Munich-Gauting, Munich, Germany; 12 Comprehensive Pneumology Center (CPC), Research Unit Lung Repair and Regeneration, Helmholtz Zentrum München, Member of the German Center for Lung Research (DZL), Munich, Germany; 13 University of Colorado, Department of Pulmonary Sciences and Critical Care Medicine, Denver, CO USA

**Keywords:** Respiration, Regeneration, Time series, Respiratory tract diseases, Translational research

## Abstract

The cell type specific sequences of transcriptional programs during lung regeneration have remained elusive. Using time-series single cell RNA-seq of the bleomycin lung injury model, we resolved transcriptional dynamics for 28 cell types. Trajectory modeling together with lineage tracing revealed that airway and alveolar stem cells converge on a unique Krt8 + transitional stem cell state during alveolar regeneration. These cells have squamous morphology, feature p53 and NFkB activation and display transcriptional features of cellular senescence. The Krt8+ state appears in several independent models of lung injury and persists in human lung fibrosis, creating a distinct cell–cell communication network with mesenchyme and macrophages during repair. We generated a model of gene regulatory programs leading to Krt8+ transitional cells and their terminal differentiation to alveolar type-1 cells. We propose that in lung fibrosis, perturbed molecular checkpoints on the way to terminal differentiation can cause aberrant persistence of regenerative intermediate stem cell states.

## Abstract (web summary)

Injury repair is characterized by the generation of transient cell states important for tissue recovery. Here, the authors present a single cell RNA-seq map of recovery from bleomycin lung injury in mice and uncover a Krt8+ transitional stem cell state that precedes the regeneration of AT1 cells and persists in human lung fibrosis.

## Introduction

Lung disease is a major health burden accounting for one in six deaths globally^1^. The lung’s large surface area is exposed to a great variety of environmental and microbial insults causing injuries to its epithelium that require a stem cell driven regenerative response. Lineage tracing studies revealed that depending on the location within the lung and the severity of injury, different stem cell populations can be engaged^2–4^. The cell-intrinsic properties and niche signals driving these processes are not well understood and likely involve tight spatiotemporal control of crosstalk with the various immune and mesenchymal cell types that are activated or recruited after injury^5,6^. Importantly, many of the functionally relevant cell states appear transiently after injury. For instance, the conversion of fibroblasts to myofibroblasts during fibrogenesis has been shown to be reversible^7^. Similarly, the recruitment of monocytes early after injury results in a continuum of macrophage states that evolve as their microenvironment changes over time^8^. This implies that functionally important cell states are limited in time and space by yet to be resolved regulatory mechanisms.

In the alveolar compartment, gas exchange is enabled by ultra-thin extensions of alveolar type-1 pneumocytes (AT1) forming the alveolar surface area. The surfactant-producing cuboidal alveolar type-2 pneumocytes (AT2) have been shown to act as alveolar stem cells by self-renewing and differentiating into squamous AT1 cells, during both homeostatic turnover and injury^9^. In very severe cases of injury with massive loss of AT2 cells, both AT1 and AT2 cells can be replenished by airway-derived stem cell populations^10–13^. The molecular details and spatiotemporal organization of such decisive signals, gene programs and pathways during recovery of the AT1 cell layer have not been resolved.

Using single-cell RNA sequencing (scRNAseq) methods it is now possible to predict the future state of individual cells based on RNA velocity^14^ and model cell fate trajectories in pseudotime^15,16^. These methods are highly complementary with traditional lineage tracing and longitudinal single-cell analysis of a dynamic system^17^, combined with computational methods is unbiased and allows for discovery in high-throughput. Furthermore, the dynamics of cell–cell communication networks can be computationally approximated from scRNAseq datasets by the integration of receptor-ligand databases^18,19^. Here, we ask if we can leverage these ideas for the problem of gene regulation during epithelial regeneration.

We chart the cell type specific gene expression trajectories in whole-lung single-cell suspensions after bleomycin induced lung injury to provide a resource of the gene expression dynamics and routes of cell–cell communication during regeneration after bleomycin induced lung injury. In this analysis we discover an intermediate alveolar epithelial cell state forming a unique cellular niche that peaks in frequency during the fibrogenic phase of tissue repair together with the appearance of myofibroblasts and M2-macrophages. Using high resolution pseudotime modeling and lineage tracing we demonstrate transcriptional convergence of airway and alveolar stem cells into the transitional stem cell state and reveal candidate transcriptional regulators. Disease relevance of the regenerative intermediate stem cell state described in this work is emerging from our observation that this cell state accumulates and persists in lung fibrosis.

## Results

### A time-resolved single cell analysis of lung regeneration

To comprehensively chart the cellular dynamics of all major cell lineages during regeneration after bleomycin-mediated acute lung injury, we collected whole-organ single cell suspensions from six time points after injury (day 3, 7, 10, 14, 21, and 28) and uninjured control lungs (PBS) with on average four replicate mice per time point. Using the Dropseq workflow^20^, we generated single cell transcriptomes from ~1000 cells per individual mouse, resulting in a final data set with 29,297 cells after quality control filtering.

**Fig. 1:**
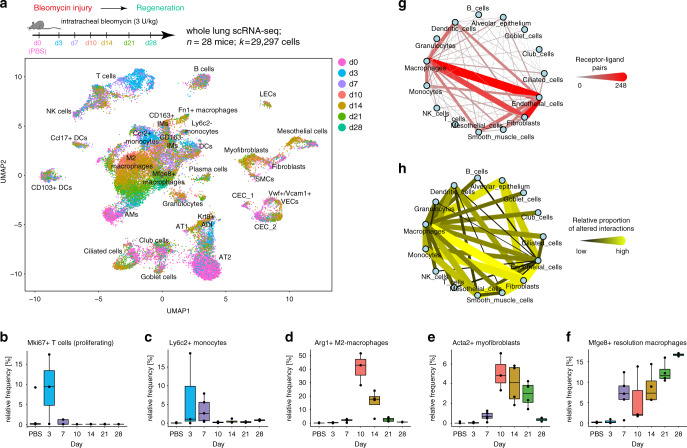
Longitudinal single cell RNA-seq reveals cell state and cell communication dynamics. **a** Single cell suspensions from whole-mouse lungs were analyzed using scRNAseq at the indicated time points after bleomycin-mediated lung injury. The color code in the UMAP embedding shows shifts of the indicated cell types in gene expression space during the regeneration time course. **b**–**f** Relative frequency of the indicated cell types relative to all other cells was calculated for individual mice at the indicated time points after injury (*n* = 4) and for PBS treated control mice (*n* = 7). The boxes represent the interquartile range, the horizontal line in the box is the median, and the whiskers represent 1.5 times the interquartile range. **g** The network shows 15 meta-cell type identities (see Supplementary Fig. 1d) and their putative communication structure. Edge weight and color illustrate the number of receptor-ligand pairs between cell types. **h** The edges represent the relative proportion of receptor-ligand pairs between cell types with altered expression after injury.

Single cell transcriptional profiles were visualized in two dimensions using the Uniform Manifold Approximation and Projection (UMAP) method^21^ (Fig. 1a). We identified 26 cell type identities that were manually annotated using canonical marker genes and previously published scRNAseq datasets of the mouse lung^22,23^ (Supplementary Fig. 1a, b; Supplementary Data 1). Most cell clusters contained cells from both conditions (Supplementary Fig. 1c) and we found good reproducibility of quality metrics across samples (Supplementary Fig. 1d). Linear discriminant analysis confirmed good agreement of cell type frequencies between conditions with 93% accuracy, demonstrating high replicability of the mouse replicates (Supplementary Fig. 1e–g, and Supplementary Fig. 2).

Cell frequency dynamics showed an expansion of T cells early after injury at day 3 (Fig. 1b), recruitment of Ly6c2+ monocytes from blood within the first weeks after injury (Fig. 1f), the appearance of Arg1; +M2-macrophages peaking at day 10 (Fig. 1c), transient formation of Acta2+ myofibroblasts (Fig. 1d; Supplementary Fig. 3), and appearance of a Mfge8+ resolution macrophage state peaking at day 28 (Fig. 1e). Subclustering macrophages revealed several distinct phenotypes at different time points (Supplementary Fig. 4a–c). Previously published bulk RNAseq signatures from lineage tracing of monocyte-derived macrophages in the bleomycin model^8^ were used to score individual cells, revealing that recruited monocytes give rise to several different macrophage identities (Supplementary Fig. 4d–g).

A total of 6660 genes showed significant changes after injury in at least one cell type (FDR < 0.1, Supplementary Data 2). The results of this analysis can be interactively explored with our webtool at github.com/theislab/LungInjuryRegeneration, which provides a user-friendly resource of gene expression changes in the whole lung during injury repair.

We constructed a putative cell–cell communication network by mapping known receptor-ligand pairs across cell types (see “Methods” for details) (Fig. 1g), and integrated longitudinal expression dynamics of receptor-ligand pairs. This analysis revealed considerable alterations in possible communication routes between macrophages and fibroblasts, as well as striking differences in communication of these cell types with the alveolar epithelium (Fig. 1h).

**Fig. 2:**
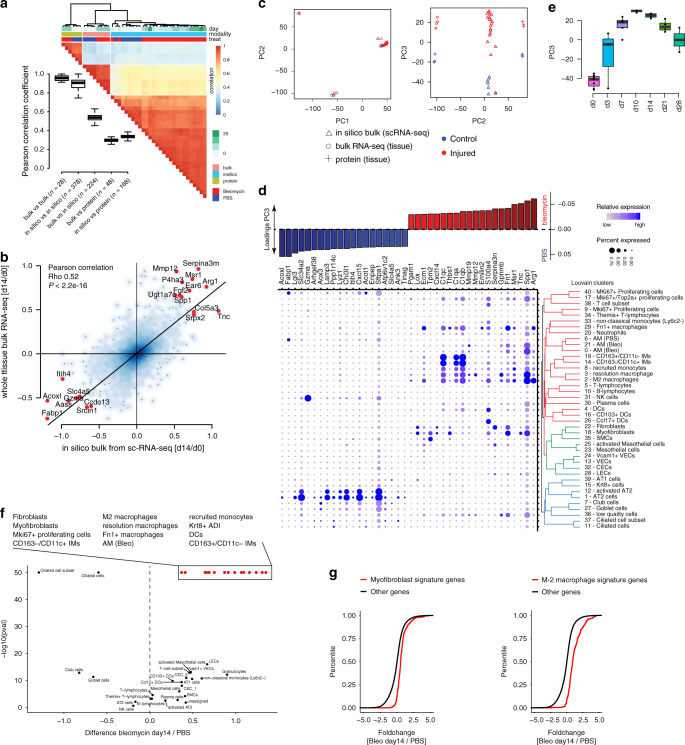
Bulk deconvolution reveals cellular source of regulated proteins and cell state frequency changes. **a** Pairwise Pearson correlation was calculated across whole lung bulk RNA-seq (bulk, *n* = 4), in silico bulk scRNA-seq (in silico, Bleo *n* = 4, PBS *n* = 7) and proteomics samples (protein, *n* = 4). Bulk and proteomics data contain samples from day-14 after bleomycin-induced injury and controls^24^. Red and blue colors indicate high and low correlation values, respectively. Columns are ordered by unsupervised hierarchical clustering. Colored bars on top of heatmap indicate time point, data modality and injury status of each sample. Boxplot displays the distribution of Pearson correlation coefficients across comparisons between various data modalities; boxes represent the interquartile range, the horizontal line the median, and the whiskers 1.5 times the interquartile range. **b** Scatter plot depicts fold changes calculated between day 0 and 14 for the bulk (*y*-axis) and in silico bulk (*x*-axis) RNAseq samples. The black line represents the Deming regression line. Top 20 genes with the highest average fold change in both modalities are highlighted. Statistical significance was assessed using Pearson correlation (*p* < 2.2e−16). **c** Data from all three modalities was integrated. The first two principal components show clustering by data modality. The third principal component separates bleomycin samples from controls across all three data modalities. Blue and red colors indicate control and bleomycin samples. **d** Barplot on top depicts genes with the highest loadings for principal component 3. **e** The box plot shows the time-resolved loading of PC3 peaking at day 10. The boxes represent the interquartile range, the horizontal line in the box is the median, and the whiskers represent 1.5 times the interquartile range (Bleo, *n* = 4 per timepoint; PBS, *n* = 7). **f** Volcano plot illustrates results from the bulk deconvolutions analysis. *X* axis indicates mean fold change of cell type markers between day 14 and PBS bulk samples. *Y* axis displays the −log10 *p*-value derived from a two-sided Kolmogorov-Smirnov test. *P*-values were limited to a minimum of 1e−50 for visualization purposes. **g** Empirical cumulative density plots show two exemplary cell types Myofibroblasts (right) and M2 macrophages (left). Red and black lines correspond to the distribution of cell type markers and all other genes, respectively.

To validate the scRNAseq data we performed extensive comparisons with our previously published bulk RNA-seq and proteomics data from day 14 after bleomycin treatment^24^. We generated in silico bulk samples by summing counts across all cells of each mouse replicate. Significant correlations were observed across all three modalities (Fig. 2a, b), and samples clustered by data modality but also injury status, cross-validating the global injury-induced expression changes (Fig. 2c). Interestingly, the shared bleomycin induced features across data modalities mostly showed cell type specific expression in the alveolar epithelium, fibroblasts and macrophages (Fig. 2d), with a peak at day 10 and resolving during the regeneration time course (Fig. 2e). To validate changes in cell type frequency observed at the cellular level, we performed bulk deconvolution analysis, testing for enrichment of cell type marker signatures in the bulk RNA-seq data. This analysis revealed cell types and states with significantly increased frequency after bleomycin injury (Fig. 2f, g).

### Unique squamous Krt8+ cells in alveolar regeneration

**Fig. 3:**
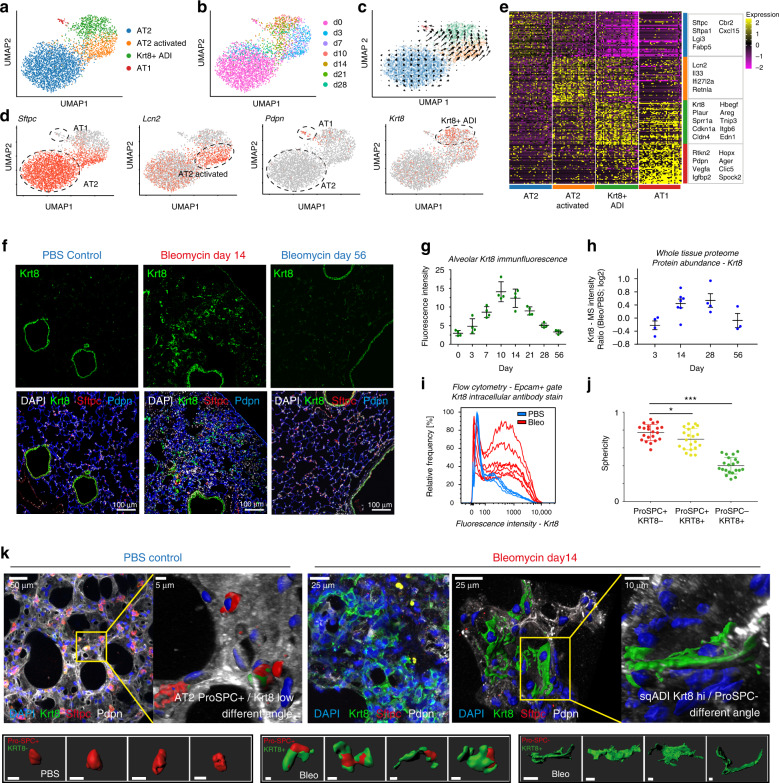
Alveolar regeneration features a transient squamous cell state marked by Krt8 expression. **a**–**d** UMAP embedding of alveolar epithelial cells shows (**a**) four distinct cell states, and (**b**) the time points of sampling, and (**c**) the RNA velocity vectors, indicating AT2 cell differentiation towards the alveolar Krt8+ cell state after bleomycin-mediated injury, and (**d**) gene expression of the indicated marker genes. **e** Heatmap of top 50 differentially expressed genes across alveolar cell states, with selected marker genes in boxes. **f** Fluorescent immunostainings from the indicated conditions show nuclei (DAPI) in white, Krt8 in green, Sftpc (AT2 cells) in red, and Pdpn (AT1 cells) in blue (scale bar 100 microns). **g** Quantification of Krt8 mean fluorescence intensity in alveolar space (excluding airways; *n* = 4 per time point, mean with SD). **h** Protein abundance of Krt8 in total lung homogenates was assessed by mass spectrometry^24^. Individual data points show log2 ratio of Krt8 MS-intensity after bleomycin injury [*n*(d3) = 4, *n*(d14) = 7, *n*(d28) = 4, *n*(d56) = 3] versus PBS control mice (*n* = 4). The mean and standard error of the mean is shown. **i** Krt8 fluorescence intensity quantified by flow cytometry in epithelial cells. PBS control (*n* = 5, blue color) and day 10 after bleomycin (*n* = 7, red color) is shown. **j** Alveolar cell sphericity analysis of 21 cells per condition revealed elongated cell shapes for alveolar Krt8+ cells in IF-stained precision cut lung slices (in **k**). Sphericity of 1 indicates round, cuboidal cells, 0 indicates flat cells. PBS, *n* = 2; Bleo, *n* = 2. One-way ANOVA with Dunnett’s post testing: *p = 0.0376, ***p < 0.0001. **k** Maximum projections of confocal z-stacks taken from immunostained 300 micron-thick precision cut lung slices (PCLS) are shown for a representative PBS control mouse and a mouse at day 14 after bleomycin injury. Nuclei (DAPI) are colored blue, Krt8 appears in green, Sftpc (AT2 cells) in red, and Pdpn (AT1 cells) in white. Image data representation stems from *n* = 5 samples. Small images below show examples taken for cell morphometric analysis (in **j**). All scale bars in small single-cell images represent 15 µm.

One of the clusters with significantly enriched frequency after injury represented a so far undescribed cell state in the alveolar epithelium, marked by high expression of Keratin-8 (Krt8) and a highly distinct set of genes. Subclustering of alveolar epithelial cells resulted in four distinct clusters (Fig. 3a), which largely represented different time points (Fig. 3b). Notably, AT1 and AT2 cells were connected by cells mainly derived from intermediate time points. We identified AT2 cells marked by Sftpc expression, and an activated AT2 state marked by injury-induced genes, such as Lcn2 and Il33 (Fig. 3d, e). The Krt8+ cells showed some transcriptional similarity to AT1 cells, however, were clearly distinct and did not highly express the canonical marker genes for AT2 and AT1 (Fig. 3d, e). To analyze a possible transition of AT2 cells to these cells we used scVelo (see Methods for details) which uses the ratio of spliced to unspliced reads to infer RNA velocities^14^ and computationally predicts the future state of individual cells. This RNA velocity analysis suggested that alveolar Krt8 high cells were derived from activated AT2 cells and might give rise to AT1 cells (Fig. 3c, d). Thus, we named the cell state Krt8+ alveolar differentiation intermediate (ADI).

Immunostainings of Krt8 in lung sections confirmed its transient de novo expression in lung parenchyma. We observed a peak of alveolar Krt8 expression around day 10–14 after injury (Fig. 3f, g; Supplementary Fig. 5a). In contrast, the uninjured control lungs and fully regenerated lungs at eight weeks after injury showed Krt8 expression only in airways (Fig. 3f). The transient burst of Krt8 protein expression was additionally validated on whole tissue level using mass spectrometry (Fig. 3h) and flow cytometry (Fig. 3i; Supplementary Fig. 5b, c).

Alveolar Krt8+ cells featured high expression of pro-fibrogenic proteins, including the low-affinity epidermal growth factor receptor ligands Areg and Hbegf, as well as the integrin Itgb6, validated by flow cytometry (Supplementary Fig. 5b, c), and immunostainings (Supplementary Fig. 5d, e). A recent report has highlighted the important role of the Yap/Taz signaling pathway in alveolar regeneration^25^. We found high levels of nuclear YAP in Krt8+ ADI cells and also some myofibroblasts, indicating active Yap/Taz signaling (Supplementary Fig. 5f, g). The expression of many Yap/Taz target genes can also be activated by TGF-beta signaling. We therefore assessed if Krt8+ ADI cells also feature high levels of phospho-SMADs and found that pSMAD2 staining in fibrotic areas after bleomycin injury specifically marked Acta2+ myofibroblasts and was surprisingly absent in Krt8+ ADI (Supplementary Fig. 5h, i).

Morphometric analysis on 300 micron-thick precision cut lung slices revealed that Krt8 is expressed only at very low levels in cuboidal AT2 cells in the uninjured lung, while in bleomycin injured lungs it is increased in still cuboidal AT2 cells expressing Sftpc and at highest levels in Sftpc negative cells with squamous morphology (Fig. 3k). In comparison to AT2 cells, the Krt8+ cells showed a significantly reduced sphericity factor and also AT2 cells with upregulated Krt8 after injury were found to assume a significantly flatter shape (Fig. 3j).

To determine if the appearance of alveolar Krt8+ ADI is specific to the bleomycin injury model, we turned to two other independent mouse models that are not based on DNA damage for the injury. Alveolar Krt8 expression was increased in a model of neonatal hypoxia and hyperoxia with Influenza type-A infection^26^ (Supplementary Fig. 6a), as well as exposure of adult mice to hyperoxia, which has been shown to preferentially kill alveolar AT1 cells^27^ (Supplementary Fig. 6b).

### A sky dive into epithelial cell transitions after injury

**Fig. 4:**
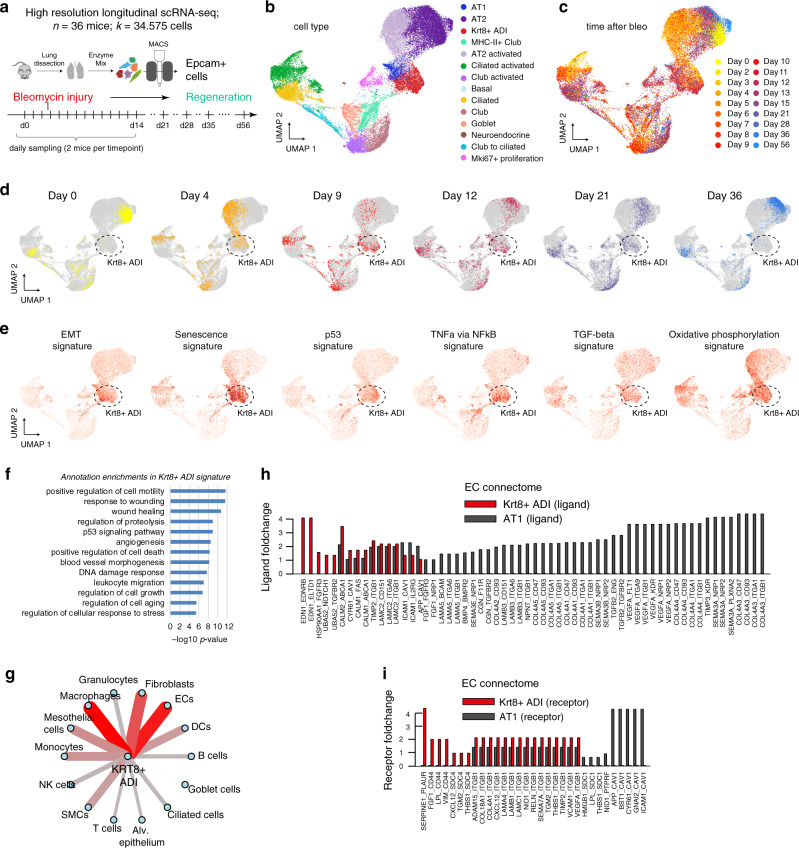
Krt8+ADI cells feature unique pathway and cell–cell communication activities. **a** A high-resolution longitudinal data set was generated by subjecting sorted cells from the epithelial compartment to scRNAseq at the 18 indicated time points. UMAP embedding displays cells colored by (**b**) cell type identity and (**c**) time point. **d** The colored dots on the UMAP illustrate densities and distribution of cells at individual time points after bleomycin injury. Note the time dependent movement of cells within the data manifold. **e** UMAP embedded visualizations of single cells colored by gene expression signature scores for the indicated pathways (MSigDB Hallmark gene sets). **f** The indicated terms were significantly enriched in the Krt8+ ADI signature compared to all other epithelial cell states. **g** The cell–cell communication network displays the number of receptor-ligand pairs between the molecular markers of the Krt8+ ADI state and all other meta cell type identities (Fig. 1). **h**, **i** The bar graphs show the average log2 fold change of either (**h**) receptors or (**i**) ligands within the endothelial cell (EC) connectome for Krt8+ ADI and AT1 cells.

To model the generation of Krt8+ ADI at higher temporal and cellular resolution we sorted EpCam+ cells and sampled single cell transcriptomes daily up to day 13. We also included later time points up to day 54 after injury to analyze the recovery of the system back to baseline with fully regenerated AT1 cells. In total, we collected 18 time points after injury using two replicate mice each (*n* = 36 mice; *k* = 34575 cells) (Fig. 4a).

Cell type identities were consistent with the first whole-lung experiment and we identified rare neuroendocrine cells and basal cells in addition (Fig. 4b; Supplementary Fig. 7). We observed gene expression changes of AT2 cells with cell state densities moving towards the Krt8+ ADI state already at early time points starting at day 2 (Fig. 4d). A continued presence of Krt8+ ADI cells was then seen until day 36 (Fig. 4d, Supplementary Fig. 7). Scoring single cells for enrichment of gene programs revealed that in comparison to the other epithelial cells, Krt8+ ADI displayed high scores for genes involved in epithelial–mesenchymal transition (EMT), cell senescence, and the p53, MYC, TNFA via NFkB, and oxidative phosphorylation pathways (Fig. 4e). All these pathways have been characterized by expression of a host of secreted factors that may promote fibrogenesis. Statistical analysis of pathway enrichment confirmed the strong and specific enrichment of genes previously associated with wound healing, angiogenesis and the p53 pathway in the Krt8+ ADI cells (Fig. 4f).

We hypothesize that the Krt8+ ADI cell state with its unique gene expression program serves important niche functions to coordinate other cell types during tissue regeneration. In the receptor-ligand database (Fig. 1) the Krt8+ ADI show their largest number of receptor-ligand pairs with fibroblasts, macrophages and (capillary) endothelial cells (Fig. 4f; Supplementary Data 6). Interestingly, in the endothelial cell (EC) connectome with Krt8+ ADI and AT1, the capillary ECs received signals via the endothelin-receptor (Ednrb) expressed on ECs via the ligand endothelin-1 (Edn1), which was specifically expressed on Krt8+ ADI and not on AT1 (Fig. 4g). Conversely, the AT1 cells displayed a large number of ligands, including Vegfa and Sema3e that bind receptors, such as Flt1 or Nrp1/2 on ECs, which were not expressed on Krt8+ ADI. Similar selective differences between Krt8+ ADI and AT1 were observed for receptors such as the urokinase plasminogen activator receptor (Plaur) specifically expressed on Krt8+ ADI but not AT1, binding to the EC-derived ligand PAI-1 (Serpine1) (Fig. 4h).

### Involvement of airway stem cells in alveolar regeneration

**Fig. 5:**
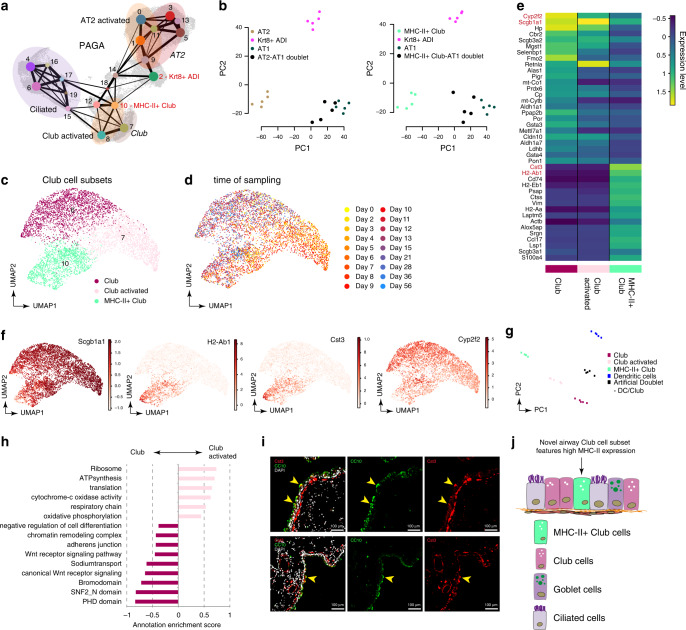
A distinct club cell state shows high connectivity to alveolar cell identities after injury. **a** The PAGA graph visualizes potential cell-type transitions and the topology of the data manifold. Nodes represent Louvain clusters and thicker edges indicate stronger connectedness between clusters. **b** Principal component analysis of artificially generated cluster-specific and doublet in silico bulk samples shows that Krt8+ ADI cells map orthogonal with respect to linear activated AT2 to AT1 (left) and MHC-II + club to AT1 (right) differentiation profiles. In silico bulk samples are colored by cluster as derived from the PAGA map in (**a**). Artificially generated doublets are colored in black. **c**, **d** The plots visualize the UMAP embedding of Club cells colored by Louvain clustering (**c**) and by time point (**d**). **e** The heatmap shows the average expression levels of marker genes across the three club cell clusters. **f** UMAP embeddings show distinct expression patterns for selected marker genes. **g** Principal component analysis of artificially generated cluster-specific and doublet in silico bulk samples shows that MHCII + club cells map orthogonal with respect to a linear connection between dendritic and club cells. Dendritic cell samples and artificially generated doublets are colored in blue and black, respectively. **h** The bar graph shows the annotation enrichment score^78^ for selected examples of gene categories with significant enrichment (FDR < 5%) in either activated Club (positive scores) or Club cells (negative scores). **i** Immunofluorescence staining of mouse airways shows CC10+ club cells (green) and Cst3+ cells (red), DAPI (white). Note the partial overlap of Cst3+/CC10+ airway cells (highlighted by yellow arrowheads). Scale bar = 100 microns; representative images from *n* = 3 bleo-treated mice. **j** Revised model of club cell heterogeneity in mouse airways.

To analyze global connectivity and potential trajectory topology in the epithelial cell state transitions we applied partition-based graph abstraction (PAGA) (Fig. 5a), which provides an interpretable graph-like map of the data manifold^28^. Interestingly, the PAGA map revealed several nodes with high connectivity between cell types that represented potential transdifferentiation bridges. In particular, we observed a subset of airway club cells (cluster 10) with connectivity to all alveolar cells including Krt8+ ADI, and an activated AT2 cell state (cluster 9) which also featured high connectivity to Krt8+ ADI. We simulated gradual differentiation intermediates by generating in silico doublets combining AT1 with cluster 10 and 9 (Fig. 5b). The simulated doublets mapped between these clusters and AT1 samples, while Krt8+ ADI cells mapped orthogonal to linear differentiation trajectories towards AT1. This demonstrates that the Krt8+ ADI state is highly distinct and does not resemble a linear gene expression intermediate from stem cells towards AT1 (Fig. 5b).

Two clusters (7 and 8) mainly represented club cells at different times after injury, which we termed club and club activated, respectively (Fig. 5c, d, h). Cluster 10, however, was highly distinct and surprisingly marked by high expression of MHC-II complex genes (e.g. H2-Ab1) and the cysteine proteinase inhibitor Cystatin-C (Cst3), which is typically co-expressed with MHC-II in dendritic cells^29^ (Fig. 5e, f). Of note, MHC-II positive club cells were not doublet artefacts as evidenced by comparison to artificially generated club and dendritic cell doublets (Fig. 5g). We additionally validated the MHC-II + club cell state using immunofluorescence of Cst3 that stained a rare subset of Scgb1a1+ airway club cells (Fig. 5i). Taken together, our data suggests the existence of a distinct cell state within the club cell lineage, marked by high expression of MHC-II genes, that features high connectivity to alveolar epithelial cell identities. Importantly, a recent report described a very similar gene signature in club-like epithelial progenitors that regenerated both AT2 and AT1 cells in the bleomycin model^30^, suggesting that we have identified the same stem cell in our data.

We occasionally found rare cells with high levels of *Krt8* expression in the alveolar space of uninjured control lungs (Supplementary Fig. 7, 10a), suggesting that the same cell state observed after injury may be a natural intermediate of homeostatic cell turnover. These pre-existing alveolar Krt8+ cells did not undergo proliferative expansion. The relative frequency of Ki67+ proliferating cells in the single cell data manifold (cluster 14) peaked at day 15 (Supplementary Fig. 9a). Counting Ki67+ cells in immunostainings confirmed the peak of cell proliferation around day 14 with a sudden drop in proliferation rates around day 28 (Supplementary Fig. 9d, e). Cell cycle regression within the proliferative cells enabled us to deconvolve cell type identity (Supplementary Fig. 9b), revealing that Krt8+ ADI cells, AT2, club, and the MHC-II + club cells all proliferated after injury (Supplementary Fig. 9c). We validated proliferating Krt8+ cells in co-immunostainings Ki67+ at day 10 after injury (Supplementary Fig. 9f). Importantly, the massive expansion of Krt8+ ADI over time happened without spiking numbers of Krt8+/Ki67+ cells preceding this (Supplementary Fig. 10b). Using tamoxifen labeling in SPC-CreERT2 and Sox2-CreERT mice we found that the rare pre-existing Krt8+ ADI cells were 80% labeled in the SPC-CreERT2 mice (Supplementary Fig. 10c–e), suggesting that these cells are derived from AT2, possibly during normal homeostatic turnover.

### Transcriptional convergence of alveolar and airway stem cells

**Fig. 6:**
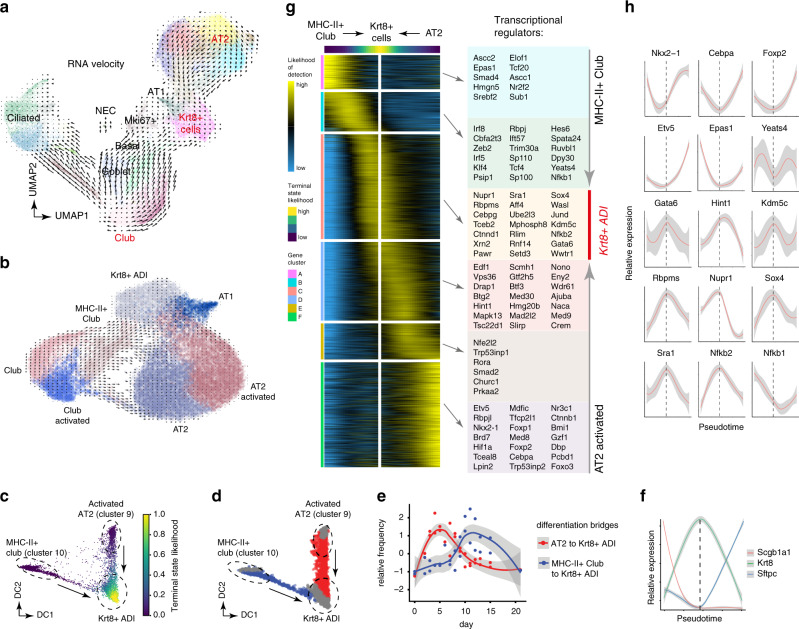
Transcriptional convergence of MHC-II+;club and AT2 cells onto the alveolar Krt8+ADI cell state. **a** Velocity plot displays the UMAP embedding colored by Louvain clusters with velocity information overlaid (arrows). **b** Velocity plot of a subset of the data only showing alveolar identities and club cell subsets. RNA velocity shows contribution of Scgb1a1+ club cells to both Krt8+ ADI and AT2 identities. **c** Diffusion map of Louvain clusters 2, 10, and 9 colored by inferred terminal state likelihood reveals two distinct transdifferentiation trajectories from activated AT2 and MHC-II + club cells towards a Krt8+ cell state. **d** Diffusion map colored by groupings derived from Gaussian Mixed Model Clustering. Red and blue colors represent AT2 and MHC-II + club cell differentiation bridges towards the Krt8+ ADIs. Grey colors represent cells at endpoints. **e** The lines indicate smoothed relative frequencies across time points of cells within the AT2 (red) and MHC-II + club cell (blue) differentiation bridges. **f** The lines illustrate smoothed expression levels of Scgb1a1, Krt8, and Sftpc across the trajectory, marking cell identities. The dashed vertical line indicates the peak of Krt8 expression. **g** The heatmap shows the gene expression patterns along the differentiation trajectory based on the inferred likelihood of detection for 3036 altered genes. **h** Line plots show the smoothed relative expression levels of selected transcriptional regulators across the converging trajectories. The dashed vertical line indicates the peak of Krt8 expression. For (**e**), (**f**), and (**h**), gray colors represent the 95% confidence interval derived from the smoothing fit.

RNA velocity vectors overlaid onto the UMAP embedding predicted transdifferentiation of club cells towards ciliated and goblet cells, which is in agreement with previous literature^2^ (Fig. 6a). Interestingly, RNA velocities also strongly suggested a dual origin of alveolar Krt8+ ADI cells from AT2 and airway cells, in particular from Scgb1a1+ club cells (Fig. 6a, b). Club cells and MHC-II+club cells show differentiation bridges towards AT2 cells and Krt8+ ADI (Fig. 6b). As MHC-II + club cells showed very high connectivity to Krt8+ ADI and were closest in the UMAP embedding, we restricted the analysis to the activated AT2, MHC-II + club and Krt8+ ADI states, and calculated terminal state likelihoods based on RNA velocities, which showed differentiation of both activated AT2 and MHC-II + airway club cells towards Krt8+ ADI (Fig. 6c). Even though MHC-II + club cells (cluster 10) showed high connectivity with alveolar cells (Fig. 5b), the data indicates that also other Scgb1a1+ club cells can give rise to alveolar cells during injury repair.

Restricting the analysis to cells “bridging” from the AT2 and MHC-II + club cells to Krt8+ ADI (Fig. 6d), we found that the AT2 conversion preceded the MHC-II + club to Krt8+ ADI differentiation by about one week (Fig. 6e). This may indicate that alveoli with surviving AT2 cells regenerate faster than alveoli with total loss of AT2 that require recruitment of distal airway stem cells. Thus, the data shows convergence of transcriptional states from distinct lineages (airway stem cells versus alveolar AT2) even at different times after injury.

We identified 3036 genes showing distinct expression patterns along these differentiation trajectories (Fig. 6f, g; Supplementary Data 4). We observed a gradual decline in expression of the Homeobox protein Nkx-2.1, critical for lung development and lung epithelial identity^31^, as well as Foxp2, which is one of the key transcriptional repressors involved in the specification and differentiation of the lung epithelium^32,33^, in both MHC-II + club and AT2 cells during conversion to Krt8+ ADI (Fig. 6h). Also, expression of the transcription factor Cebpa with important functions in lung development and maintenance of both club and AT2 cell identity^34–36^ reached a minimum at the Krt8+ ADI state. AT2 cell conversion into Krt8+ ADI was marked by a drastic reduction of the transcription factor Etv5, which has been shown to be essential for the maintenance of AT2 cells^37^ (Fig. 6h). Conversely, the differentiation towards the Krt8+ ADI signature expression was characterized by a gradual increase in one of the master regulators of AT1 cell differentiation Gata6^38,39^ in both MHC-II + club and AT2 cells. Both trajectories converged on a large number of alveolar Krt8+ ADI specific genes representing distinct pathways (Fig. 4) and their transcriptional regulators, including the stress-induced p53 interactor Nupr1, a master regulator of epithelial to mesenchymal transition Sox4, and many other genes, including chromatin remodeling factors such as the histone demethylase Kdm5c (Fig. 6h). To validate our findings we re-analysed the scRNAseq data set from whole-lung suspensions (Fig. 1), which confirmed differentiation of AT2 cells onto the Krt8+ ADI state and contribution of airway cells to alveolar fates (Supplementary Fig. 10).

**Fig. 7:**
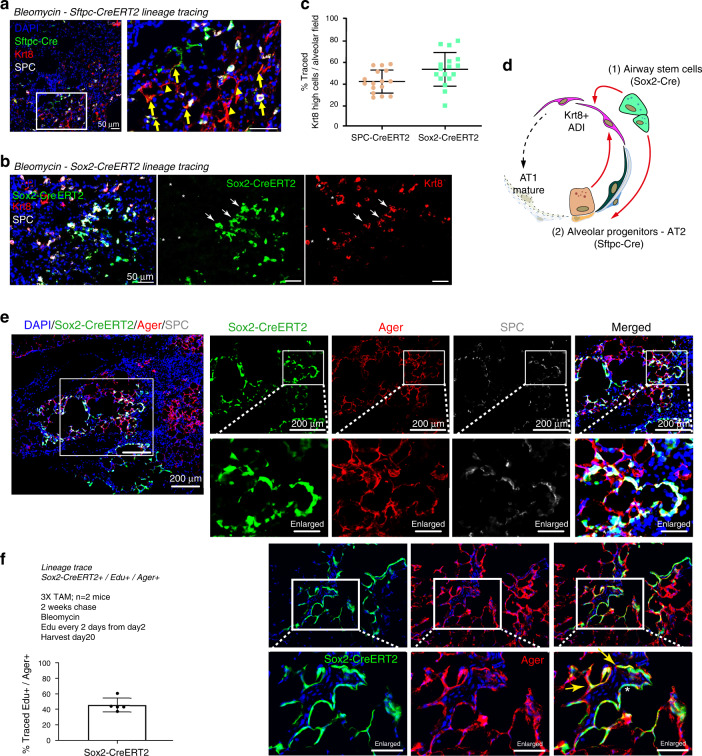
Lineage tracing validates dual origin of Krt8+ADI. **a**, **b** Immunostainings of Krt8 and Sftpc (SPC) in (**a**) Sftpc-CreERT2-labeled mice (*n* = 2) and **b** Sox2-CreERT2-labeled mice (*n* = 2; lobes analyzed per mouse: *n* = 3). Arrows indicate lineage positive and stars lineage negative Krt8+ ADI. **c** Quantification of lineage labeled alveolar cells with high Krt8 expression. Each point in the graph represents a large region (at least 1.2 mm^2^ area) and cells from at least three lobes/mouse at two different levels (>100 μm apart) were analyzed in 16 fields of view (Sftpc-CreERT2: *n* cells = 1382; Sox2-CreERT2: *n* cells = 1833). **d** Lineage tracing experiments validate the scRNAseq experiments and show convergence of distinct alveolar progenitors into Krt8+ ADI. **e** Immunostainings of the AT1 marker Ager and the AT2 marker SPC with the Sox2-CreERT2 lineage label at day 14 after bleomycin. Heavily injured regions show only little endogenous SPC+ cells but also Sox2-traced Ager+ and SPC + cells. The experiment was performed on *n* = 2 mice and *n* = 3 lobes/mouse were analyzed. Flat lineage labeled cells can be observed Ager+ (yellow arrow) and Ager− (asterisk). **f** Sox2-CreERT2+/Ager+ AT1 cells that previously proliferated upon bleomycin injury were quantified using the indicated EdU pulse chase labeling strategy. The percentage of EdU chased and lineage labeled AT1 cells is shown. Each dot represents cell counts from at least 2 large regions from two mice, *n*(mice) = 2; data represented with mean and SD.

To experimentally validate this computational analysis, we used Sftpc-CreERT2 (AT2 cells) and Sox2-CreERT2 (airway cells) reporter mice to trace the origin of Krt8+ ADI cells back to these lineages. In the quantification of these two independent lineage tracing experiments (Fig. 7a, b), we observed that approximately half of the alveolar Krt8+ ADI cells were derived from either Sftpc-CreERT2 or Sox2-CreERT2+ airway cells in the bleomycin model (Fig. 7c, d). Of note, Sox2-CreERT2+ airway cells gave rise to both Sftpc+ and Sftpc- cells (Fig. 7b), Ager+ (AT1 marker) and Ager- squamous cells covering alveolar surfaces (Fig. 7e). We found increased contribution of Sox2-lineage labeled cells in severely injured areas. Using an EdU pulse chase labeling strategy we chased proliferating cells every other day for 20 days and counted Sox2-CreERT2+/Ager+ cells, revealing that around 40% of newly formed AT1 cells were airway derived (Fig. 7f; Supplementary Fig. 10f). In conclusion, the lineage tracing data confirms a dual origin of Krt8+ ADI cells and substantiates our prediction of a substantial contribution of airway derived stem cells in alveolar regeneration after bleomycin injury.

### Cell state trajectory model of AT1 cell regeneration

**Fig. 8:**
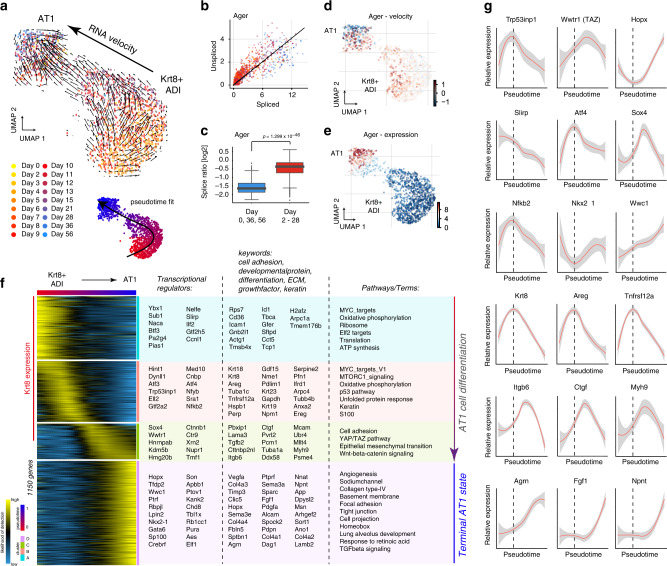
Terminal differentiation trajectory modeling of Krt8+ADI to AT1. **a** Velocity plot displays the UMAP embedding colored by time point with velocity information overlaid (arrows), indicating terminal differentiation of Krt8+ ADI into AT1 cells. **b** The velocity phase plot shows the number of spliced and unspliced reads of the AT1 marker Ager for each cell (points) on the *X* and *Y* axes, respectively. Cells are colored by time point and the black line represents the linear steady-state fit. Cells above and below the diagonal are predicted to be in inductive or repressive states, respectively. **c** The Boxplot shows the log2 ratio of unspliced over spliced Ager reads for days 0, 36 and 56 (blue, *n* = 100 cells) and all other time points (red, *n* = 1193 cells). To avoid division by zero, one was added to both counts. Statistical significance was assessed by using Wilcoxon rank-sum test (two-sided). The boxes represent the interquartile range, the horizontal line in the box is the median, and the whiskers represent 1.5 times the interquartile range. UMAP embedding colored by Ager velocity (**d**) and expression (**e**) displays a gradual increase along the inferred trajectory. **f** The heatmap shows the gene expression patterns across the differentiation trajectory for 1150 altered genes. **g** The line plots illustrate smoothed expression across the differentiation trajectory for a number of exemplary genes. Gray colors represent the confidence interval derived from the smoothing fit. The dotted line indicates the peak of Krt8 expression.

Analysis of RNA velocity information within the subset of Krt8+ ADI and AT1 cells indicated differentiation of Krt8+ ADI toward AT1 cells (Fig. 8a). The ratio of spliced and unspliced reads revealed gradual induction of transcription of the AT1 cell marker Ager in Krt8+ ADI cells around day 14 (Fig. 8b). Days 0, 36 and 56 representing a baseline with mature AT1 cells contained a significantly lower ratio of unspliced over spliced Ager reads compared to all other time points (Fig. 8c; Wilcoxon Rank Sum test, *P* < 1e-46). A gradual decrease in Ager mRNA velocity (Fig. 8d), was reflected with a gradual increase of Ager expression (Fig. 8e). Using this information, we modeled a pseudotime trajectory and determined gene expression dynamics for 1150 significantly regulated genes along the putative Krt8+ ADI to AT1 transition (Fig. 8f; Supplementary Data 5).

The differentiation trajectory was split in four phases that were marked by distinct sets of transcriptional regulators, developmental genes and signaling pathways (Fig. 8f, g). The initial phase was marked by genes and pathways consistent with cell growth after exit from the cell cycle (e.g. MYC targets). This was followed by the induction of stress-related signaling pathways, such as the p53 pathway and the unfolded protein response pathway, featuring increased expression of the corresponding transcriptional regulators such as Trp53inp1 and Atf4, and the peak of Krt8 expression (Fig. 8f, g). Next, a critical pre-AT1 stage was marked by the downregulation of the Krt8 signature and the induction of a gene expression program with similarities to the epithelial to mesenchymal transition (EMT), together with one of its master regulators Sox4^40^ (Fig. 8g). We further observed pre-AT1 specific expression of important transcriptional regulators such as TAZ (Wwtr1) and beta-catenin (Ctnnb1), and pro-fibrogenic proteins such as integrin beta-6 (Itgb6), and connective tissue growth factor (Ctgf). The non-muscle myosin heavy chain IIa (Myh9) also peaked in pre-AT1 cells, suggesting important additional cytoskeletal rearrangements and increased cell contractility in the already squamous Krt8+ ADI cells in the final steps of maturation towards AT1 cells (Fig. 8g).

Terminally differentiated AT1 cells were characterized by high expression of the transcription factors Hopx, Gata6 and Wwc1, as well as a large number of developmentally important factors, including extracellular matrix proteins and growth factors, such as Fgf1, Npnt and Agrn (Fig. 8g). It has long been noted that isolated AT2 cells spontaneously drift toward AT1 fate in vitro, suggesting that plasticity may be a cell-intrinsic property and that AT2 cell identity in vivo is actively maintained by niche signals. Interestingly, during a five-day AT2 to AT1 in vitro differentiation, Krt8 protein levels were shown to be highest at day 3, followed by the AT1 marker Pdpn peaking later at day 5^41^. We repeated this experiment and subjected isolated AT2 cells to inhibition of Wnt/β-catenin/TCF-mediated transcription, which significantly reduced the induction of Krt8 expression and levels of the AT1 cell marker Pdpn in comparison to controls (Supplementary Fig. 12, Supplementary Fig. 13).

### Aberrant persistence of Krt8+ ADI is linked to fibrosis

**Fig. 9:**
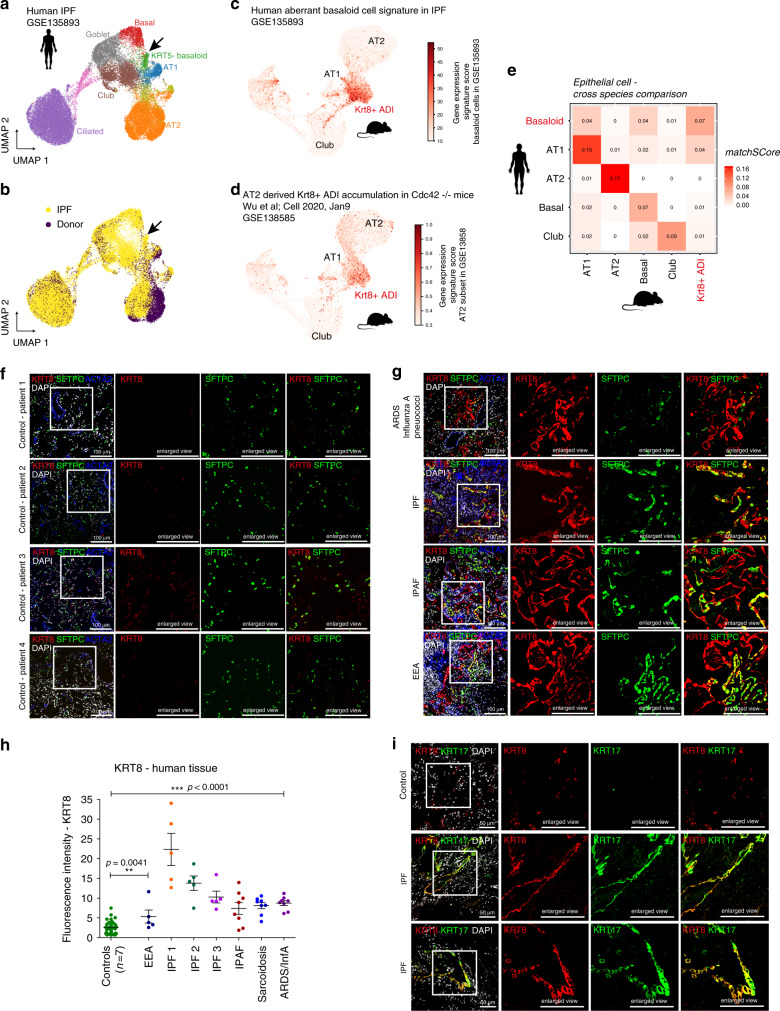
Cells similar to Krt8+ADI persist in a mouse model of progressive lung fibrosis and human disease. **a**, **b** Re-analysis of human lung fibrosis single cell data from GSE135893 for epithelial cells only. The indicated cell type identities (**a**) and disease status (**b**) show a relative increase of airway epithelial cell types in lung fibrosis (IPF) and appearance of a disease specific cell state termed aberrant KRT5−/KRT17+ basaloid cell (arrow)^42,45,79^. **c**, **d** The indicated human (**c**) and mouse (**d**) gene signatures downloaded from the Gene Expression Omnibus were scored on single cells in our mouse epithelial data manifold. Higher scores indicate higher similarity in gene expression to the indicated signatures. **e** The matchScore matrix shows the degree of similarity of the indicated cell state signatures across species. **f** FFPE sections from non-fibrotic controls were stained against KRT8 (red), SFTPC (green), and ACTA2 (blue). Scale bar = 100 microns. **g** Human lung tissue sections were stained as in **f**, revealing pronounced KRT8 expression at the site of acutely injured lesions (ARDS diagnosis) and fibrotic regions of ILD patient lungs (IPAF, IPF and EAA diagnosis). Scale bar = 100 microns. **h** Fluorescence intensity of KRT8 stainings was quantified from representative areas of control tissue [*n*(patients) = 7, *n*(areas) = 36], EEA tissue [*n*(patients) = 1, *n*(areas) = 5], IPF tissue [*n*(patients) = 3, *n*(areas per single patient) = 5], IPAF tissue [*n*(patients) = 1, *n*(areas) = 8], Sarcoidosis tissue [*n*(patients) = 1, *n*(areas) = 8], and ARDS tissue [*n*(patients) = 1, *n*(areas) = 8]. One-way ANOVA statistical analysis: ***p < 0.0001, **p = 0.0041. **i** FFPE sections from non-fibrotic controls or IPF patients were stained against KRT8 (red) and KRT17 (green). Scale bar = 50 microns; representative images from 2x IPF patients and 2x controls.

We here identified and characterized the transient appearance of Krt8+ ADI cells during lung regeneration. Single cell analysis of human lung fibrosis recently identified a disease specific cell state that was termed aberrant basaloid cell (KRT17+/KRT5-) based on some similarities to airway basal cells^42,43^. It is currently unclear if these cells are indeed airway derived or could represent stem cells undergoing alveolar repair. We re-analysed available human single cell data to extract a full gene expression signature characterizing KRT5−/KRT17+ human basaloid cells (Fig. 9a, b). Scoring the human basaloid cell signature on single cells in the mouse data manifold revealed that Krt8+ ADI cells described in this work are very similar to KRT5-/KRT17+ cells in IPF (Fig. 9c). A systematic cross-species comparison of epithelial cell state identities confirmed that human KRT5−/KRT17+ basaloid cells are most closely related to mouse Krt8+ ADI (Fig. 9e).

A recent landmark study showed that blocking alveolar stem cell differentiation, through deletion of the RhoGTPase Cdc42 in a model of pneumonectomy induced regeneration, leads to the accumulation and persistence of a unique AT2 derived cell state. These mice have progressive lung fibrosis with the typical periphery-to-center pattern of disease progression as seen in IPF patients^44^. Using quantitative comparisons of the gene expression signatures measured in this study we found that this AT2 derived cell state is also very similar if not identical to the Krt8+ ADI cells discovered by us (Fig. 9d), suggesting that persistence of Krt8+ ADI may directly mediate progressive lung fibrosis.

To further validate that KRT8+ alveolar cells can also be observed in human acute lung injury and chronic lung disease associated with alveolar injury, we stained human tissue sections and did not detect any expression of KRT8 in the alveolar space of non-injured control lungs (*n* = 7; Fig. 9f). In sharp contrast, we observed very strong alveolar *KRT8* expression in human acute respiratory distress syndrome (ARDS, *n* = 2) caused by Influenza-A and pneumococcal infection and interstitial lung disease patients with various diagnoses (*n* = 5; Fig. 9g, h). Finally, we also co-stained KRT8 with KRT17 and observed co-expression in both flat epithelial cells and bronchiolized epithelia in fibrotic areas but not in controls (Fig. 9i).

## Discussion

**Fig. 10:**
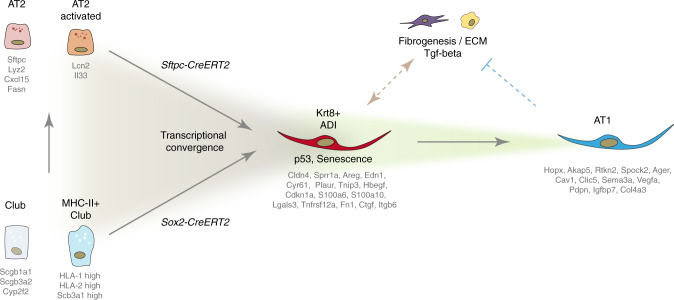
A revised model of alveolar regeneration. We identify convergence of alveolar and airway stem cells on an injury-induced transitional cell state characterized by a unique transcriptional signature, including high levels of Krt8 expression, that precedes the regeneration of AT1 cells. In this process, stem cells lose cell identity genes, gain specific gene programs including p53 and NFkB target genes, and undergo a drastic change in shape towards a squamous morphology. Krt8+ ADI cells feature a highly distinct connectome of receptor-ligand pairs with endothelial cells, fibroblasts, and macrophages. The Krt8+ ADI cell state persists in models of progressive lung fibrosis and human IPF patients, suggesting that the cell state transitions described in this work are coordinated in space and time by cell intrinsic and tissue niche checkpoints that may be derailed in disease.

In this work, we describe the dynamics of mouse lung regeneration at single cell resolution and discover the transcriptional convergence of airway and alveolar stem cells to a Krt8+ transitional stem cell state that precedes the regeneration of AT1 cells (Fig. 10). The discovery of Krt8+ ADI cells in several independent mouse lung injury models and human lung fibrosis sheds a new light on reports of EMT^45^, senescence and p53 activation^46–49^ in lung injury, repair and fibrosis. We conceptualize these observations with the appearance of this transient stem cell derived Krt8+ ADI state with its unique transcriptomic signature. Using the power of pseudotemporal modeling^15,16,50^ we analyze gene regulation during stem cell differentiation, laying out the sequence of gene programs and transcriptional regulators. Our cell state trajectory model was validated by correspondence with the real time points of sampling, RNA velocities of individual cells and lineage tracing experiments. The receptor-ligand analysis revealed potential routes of cell–cell communication and their dynamics over time. All data and code is freely available at our interactive webtool and github repository (github.com/theislab/LungInjuryRegeneration).

Even though the Krt8+ ADI gene programs resemble features found in EMT, we do not see conversion of epithelial cells to anything similar to fibroblasts. It seems that we rather see an overlap in gene expression patterns between cells undergoing genuine epithelial–mesenchymal transition (e.g. neural crest cells) and airway and alveolar stem cells changing their morphology. Morphologically, the terminal differentiation of AT1 cells in development has been shown to occur via a non-proliferative two-step process of cell flattening and cell folding^51^. We have shown that Krt8+ ADI cells in adult regeneration feature mostly squamous morphology and may thus correspond with this first phase of cell flattening. In the developmental cell folding phase, AT1 cells increase their size ten-fold to span multiple alveoli and establish the honeycomb alveolar structure in coordination with myofibroblasts and capillary vessels^51^. In this process, AT1 cells express a large number of morphogens, such as *Vegfa* and semaphorins that stimulate angiogenesis and thus likely play an active signaling role in the coordination of alveolar morphogenesis. We confirm the specific expression of these morphogens only in mature AT1 in our study and show in contrast that Krt8+ ADI express a distinct set of morphogens, including Endothelin-1 (Edn1) that likely serves the paracrine stimulation of capillary endothelial cells after injury.

In lung development, the generation of the distal epithelium has been proposed to be driven by a bipotent progenitor co-expressing both AT1 and AT2 markers^52^. Additionally, a recent scRNAseq analysis of the mouse lung epithelium at birth identified a similar AT1/AT2 cluster that may be interpreted as a bipotent progenitor state^53^. In our preliminary analysis, both published developmental signatures do not correspond well to the injury induced Krt8+ ADI signature. Additional experiments will be needed to better understand the differences of epithelial lineage trajectories in lung development versus adult homeostasis and regeneration. Interestingly, we do find rare Krt8 high alveolar cells in the parenchyma of the normal adult mouse lung. These cells are largely lineage labeled in the SPC-CreERT2 analysis but not in Sox2-CreERT2 labeling, suggesting that rare Krt8 high cells in normal homeostasis are derived from AT2 and are possibly a naturally occuring intermediate en route to AT1. We show that upon injury the bulk of AT2 cells and also airway cells differentiate into Krt8+ ADI, producing high frequencies of these cells without massive proliferation of rare stem cells at early time points.

The Krt8+ ADI cells display a highly distinct receptor-ligand connectome with mesenchyme and macrophages, and are a specific source of pro-fibrogenic factors such as *Ctgf, Itgb6, Areg, Hbegf, Edn1*, *and Lgals3*, all of which are antifibrotic targets that have been tested in pre-clinical and clinical studies. Thus, the availability of these factors (we have validated Areg, Hbegf, and Itgb6 on protein level) during the fibrogenic phase around 10 days after injury is likely dependent on the Krt8+ ADI cell state, while its transient nature elegantly enables the system to temporally limit their expression. Many pathways that peaked in the Krt8+ ADI state represent environmental stress- and inflammation-induced gene programs, represented by their transcriptional master regulators, including Trp53, Atf3/4, Nupr1, Hif1a, NFkB and TGF-beta. The proliferation of AT2 cells after lung injury involves Il1-beta and Tnf-alpha driven NFkB activation and accordingly was lost in AT2-specific IL1-receptor knock-out mice^54^, which provides a molecular link between inflammation and epithelial regeneration that is consistent with our results. We propose that inflammatory stimuli can promote cell plasticity by inducing epithelial cell states with a higher susceptibility for alternative fate programs.

Our trajectory model predicts distinct transcriptional regulators as candidate switch points in terminal AT1 differentiation, including TAZ (Wwtr1), Sox4 and beta-catenin (Ctnnb1). The mechanistic importance of Wwtr1 (YAP/TAZ - Hippo pathway) in AT2 to AT1 transdifferentiation has recently been demonstrated by using small molecule inhibition and conditional knock-out in mouse lung organoids and in vivo injury experiments^25,55^. An important role of beta-catenin and the canonical Wnt signaling pathways has been suggested based on in vitro differentiation of isolated AT2 cells^41^. Moreover, the TGF-beta pathway has been proposed to mediate cell cycle arrest in AT2 cells followed by transdifferentiation into AT1 cells^56^. Here, we found high Yap/Taz activity in Krt8+ ADI cells using immunostainings and reduced formation of Krt8+ ADI and AT1 cell states upon Wnt/beta-catenin inhibition in vitro. A functional role of *Sox4* in switching towards AT1 fate as suggested by our model awaits experimental validation.

Various potential stem cell sources for AT1 cells after injury have been described, including AT2 cells, bronchioalveolar stem cells (BASC) and p63(+)Krt5(+) distal airway stem cells (DASC). We did not see an important contribution of Krt5+ cells in our data, however, the Sox2-CreERT2 lineage tracing confirmed that after bleomycin injury a substantial fraction of Krt8+ ADI (and AT1/AT2) was derived from airway cells. We found that Scgb1a1+ club cells give rise to both AT2 and Krt8+ ADI, with a MHC-II+ subset of club cells showing a particularly strong connectivity with alveolar cell identity after injury. Comparing the signature of these MHC-II club cells with the recently described H2-K1-high epithelial progenitors^30^ suggests that these cells are identical. Strong connectivity in PAGA analysis and the observed continuous trajectories in UMAP space indicate direct conversion of airway stem cells into Krt8+ ADI, as presented in our model. However, we cannot formally exclude the possibility that airway stem cells initially give rise to AT2 and subsequently differentiate towards Krt8+ ADI. Interestingly, we find that direct AT2 cell differentiation to Krt8+ ADI precedes the differentiation of airway stem cells, suggesting that these two processes happen at different locations, possibly reflecting heterogeneity in the local severity of injury and AT2 cell survival.

In idiopathic pulmonary fibrosis patients, the aberrant activity of p53, TGF-beta, Hippo and Wnt pathway genes has been reported^57^, and a p53/p21 mediated cellular senescence program in AT2 cells, which is also reflected in the Krt8+ ADI signature, was recently proposed to drive progressive lung fibrosis in mice^46^. Furthermore, local hypoxia signaling has been implicated in dysplastic abnormal epithelial barriers^58^, which we suggest may represent an accumulation of transitional or aberrant cell states blocked in their commitment towards AT2/AT1 cell fate. Our analysis shows that the transcriptional signature of KRT5−/KRT17+ basaloid cells^45^ in IPF tissues is highly similar to the Krt8+ ADI described here. Based on these findings we propose that IPF in particular and chronic lung diseases in general may be rooted in defective molecular cell differentiation checkpoints that lead to aberrant persistence of (normally transient) regenerative intermediate cell states. Indeed, our quantitative analysis demonstrates that the Krt8+ ADI state is identical to a cell state that accumulates and persists in mice with AT2 cell specific deletion of the Rho GTPase Cdc42, which leads to progressive fibrosis similar to IPF after pneumonectomy^44^. Thus, the defective terminal differentiation of stem cells into AT1 may be a key event in pathogenesis of progressive fibrosis in IPF patients. Future therapeutic approaches may specifically aim at (re)programming Krt8+ ADI into AT1 to avoid self-amplifying paracrine feedback loops in tissue regions that are still in the early stage of disease progression.

## Methods

### Mouse experiments-bleomycin treatment

Pathogen-free female C57BL/6J mice were purchased from Charles River Germany and maintained at the appropriate biosafety level at constant temperature and humidity with a 12 h light cycle. Animals were allowed food and water ad libitum. Animal handling, bleomycin/PBS administration, and organ withdrawal were performed in accordance with the governmental and international guidelines and ethical oversight by the local government for the administrative region of Upper Bavaria (Germany), registered under 55.2-1-54-2532-130-2014 and ROB-55.2-2532.Vet_02-16-208.

### Human tissues

Resected lung tissue and lung explant material was obtained from the CPC-M bioArchive at the Comprehensive Pneumology Center (CPC), Munich. ILD diagnosed lung tissue (*n* = 6) is derived from lung explant material obtained during lung transplantation, reflecting non-resolving end-stage fibrotic disease. Healthy control tissue (*n* = 7) was derived from tumor resection in non-chronic lung disease (CLD) patients. The tissue section from a patient with ARDS (*n* = 2) has been provided by the Institute of Pathology, Ludwigs Maximilians University, Munich.

All participants gave written informed consent; the study was approved by the local ethics committee of the Ludwig Maximilians University, Munich, Germany (#333-10).

### Experimental design and animal treatment

Mice were divided randomly into two groups: (A) saline-only (PBS), or (B) bleomycin (Bleo). Lung injury and pulmonary fibrosis were induced by single-dose administration of bleomycin hydrochloride (Sigma Aldrich, Germany), which was dissolved in sterile PBS and given at 2 U/kg (oropharyngeal instillation) and 3U/kg (intratracheal instillation) bodyweight. The control group was treated with sterile PBS only. Mice were sacrificed at designated time points (days 1–14, 21, 28, 35, 56) after instillation. Treated animals were continuously under strict observation with respect to phenotypic changes, abnormal behavior and signs of body weight loss.

### Generation of single cell suspensions for whole-lung tissue

Lung single cell suspensions were generated as previously described^24^. Briefly, after euthanasia, lung tissue was perfused with sterile saline through the heart and the right lung was tied off at the main bronchus. The left lung lobe was subsequently filled with 4% paraformaldehyde for later histologic analysis. Right lung lobes were removed, minced (tissue pieces at ~1 mm^2^), and transferred for mild enzymatic digestion for 20–30 min at 37 °C in an enzymatic mix containing dispase (50 caseinolytic U/ml), collagenase (2 mg/ml), elastase (1 mg/ml), and DNase (30 µg/ml). Single cells were harvested by straining the digested tissue suspension through a 40 micron mesh. After centrifugation at 300 *g* for 5 min, single cells were taken up in 1 ml of PBS (supplemented with 10% fetal calf serum), counted and critically assessed for single cell separation and overall cell viability. For Dropseq, cells were aliquoted in PBS supplemented with 0.04% of bovine serum albumin at a final concentration of 100 cells/µl.

### Production of microfluidic devices for Dropseq

Microfluidic devices needed for scRNAseq using the Dropseq platform were fabricated by means of standard soft lithography. In brief, by using photolithography, a polydimethylsiloxane (PDMS) master mold for the Dropseq device design (CAD file available as a download from: http://mccarrolllab.org/dropseq/) was fabricated from a SU-8 photoresist (MicroChem, USA), and spin-coated on a 3″ silicon wafer to generate 125 μm-thick uniform layers. Afterwards, the master mold was filled with a 10:1 mixture of base to curing agent of the PDMS kit Sylgard 184 (Dow Corning, USA) and left at 60 °C in an oven for 4 h to crosslink the PDMS. After crosslinking, the PDMS replica was cut and peeled off from the master mold, as well as all necessary inlets/outlets for tubing connection were made in it using a 1 mm puncher. Next, the replica was sealed with a 2″ × 3″ microscopic slide, after the treatment of both in O_2_ plasma. The assembled microfluidic device was treated with Aquapel (Pittsburgh Glass Works, USA) to make all inner surfaces evenly hydrophobic.

### Single cell RNA-sequencing using Dropseq

Dropseq experiments were performed according to the original protocols^24,27^. Using the microfluidic device, single cells (100/µl) were co-encapsulated in droplets with barcoded beads (120/µl, purchased from ChemGenes Corporation, Wilmington, MA) at rates of 4000 µl/h. Droplet emulsions were collected for 10–20 min/each prior to droplet breakage by perfluorooctanol (Sigma-Aldrich). After breakage, beads were harvested and the hybridized mRNA transcripts reverse transcribed (Maxima RT, Thermo Fisher; Template-switch oligonucleotide primer: AAGCAGTGGTATCAACGCAGAGTGAATrGrGrG (50 μM)). Unused primers were removed by the addition of exonuclease I (New England Biolabs), following which, beads were washed, counted, and aliquoted for pre-amplification (2000 beads/reaction, equals ca. 100 cells/reaction) with 12 PCR cycles (Smart PCR primer: AAGCAGTGGTATCAACGCAGAGT (100 μM), 2x KAPA HiFi Hotstart Ready-mix (KAPA Biosystems), cycle conditions: 3 min 95 °C, 4 cycles of 20 s 98 °C, 45 s 65 °C, 3 min 72 °C, followed by 8 cycles of 20 s 98 °C, 20 s 67 °C, 3 min 72 °C, then 5 min at 72 °C)^27^. PCR products of each sample were pooled and purified twice by 0.6x clean-up beads (CleanNA), following the manufacturer’s instructions. Prior to tagmentation, complementary DNA (cDNA) samples were loaded on a DNA High Sensitivity Chip on the 2100 Bioanalyzer (Agilent) to ensure transcript integrity, purity, and amount. For each sample, 1 ng of pre-amplified cDNA from an estimated 1000 cells was tagmented by Nextera XT (Illumina) with a custom P5-primer (Integrated DNA Technologies; primer sequence: AATGATACGGCGACCACCGAGATCTACACGCCTGTCCGCGGAAGCAGTGGTATCAACGCAGAGT*A*C (10 μM)). Single-cell libraries were sequenced in a 100 bp paired-end run on the Illumina HiSeq4000 using 0.2 nM denatured sample and 5% PhiX spike-in. For priming of read 1, 0.5 μM Read1CustSeqB (primer sequence: GCCTGTCCGCGGAAGCAGTGGTATCAACGCAGAGTAC) was used.

Quality metrics, including the number of unique molecular identifiers (UMI), genes detected per cell and reads aligned to the mouse genome were comparable across all mice (Supplementary Fig. 1). Every time point was analyzed together with control mice that were instilled with phosphate-buffered saline (PBS). UMI-based counting of mRNA copies was used to determine differential gene expression between single cells. We used the six batches of PBS control mice to exclude dominant batch effects observing very good overlap across mouse samples (Silhouette coefficient: −0.08) (Supplementary Fig. 1).

### Processing of the whole-lung data set

For the whole-lung data set, the Dropseq computational pipeline was used (version 2.0) as described by Macosko et al.^20^. Briefly, STAR (version 2.5.2a) was used for mapping^59^. Reads were aligned to the mm10 reference genome (provided by the Dropseq group, GSE63269). For barcode filtering, we excluded barcodes with <200 detected genes. As 1000 cells were expected per sample, the first 1200 cells were used before further filtering. A high proportion (>10%) of transcript counts derived from mitochondria-encoded genes may indicate low cell quality, and we removed these unqualified cells from downstream analysis. Cells with a high number of UMI counts may represent doublets, thus only cells with less than 5000 UMIs were used in downstream analysis.

### Analysis of the whole-lung data set

The computational analysis of the whole-lung data set was largely performed using the R package Seurat^60^. Count matrices were merged using Seurat version 2.3. The merged expression matrix was normalized using the Seurat NormalizeData() function. To mitigate the effects of unwanted sources of cell-to-cell variation, we regressed out the number of UMI counts using the Seurat function ScaleData(). Highly variable genes were calculated per sample, selecting the top 7000 genes with a mean expression between 0.01 and 8. After excluding homologs of known cell-cycle marker genes^61^, a total of 18893 genes were subjected to independent component analysis. The first 50 independent components were used as input to the FindClusters() function with the ‘resolution’ parameter set to two and the RunUMAP() function with the “n_neighbors” parameter set to ten.

*Multi-omic data integration*: to confirm global expression changes observed at the single-cell level, we integrated previously published bulk RNAseq and proteomics data obtained from whole-mouse lungs 14 days after bleomycin-induced injury and controls^24^. Multi-omic data integration was performed following previous work^23,24^. Briefly, in silico bulk samples were generated by summing all counts within a mouse sample. Both the in silico bulk and whole-lung tissue bulk data were normalized using the voom() function of the limma R package^62^. Next, in silico bulk, whole-lung tissue bulk, and proteomics data were merged on a set of genes present in all three data sets and quantile normalized. This merged and quantile normalized expression matrix was then subjected to principal component analysis (PCA).

*Bulk deconvolution analysis*: to interpret the expression changes observed in the bulk RNAseq data at the cellular level and to validate the cell type frequency changes observed at the single cell level, we performed deconvolution analysis. Fold changes between the bleomycin model at day 14 and controls were obtained from Table EV4 from Schiller et al.^24^. For each cell type, marker genes with average fold change greater than zero and adjusted *p-*value < 0.25 were tested for enrichment in the fold changes by comparison to all other genes using the Kolmogorov-Smirnov test. For visualization purposes the minimum *p*-value was set to 1e-50.

*Discovery of cell type identity marker genes*: to identify cluster-specific marker genes, the Seurat FindAllMarkers() function was applied, restricted to genes detected in more than 10% of cells and with an average fold change difference of 0.25 or more. Based on these derived marker genes and manual curation we assigned all clusters to cell type and meta-cell type identities (Supplementary Fig. 1d). Cell type frequencies were calculated by dividing the number of cells annotated to a specific cell type identity, by the total number of cells for each mouse sample. In droplet-based scRNAseq data, background mRNA contamination by the so-called “ambient RNAs” is frequently observed. These mRNAs are believed to stem from dying cells which release their content upon cell lysis. This contamination is distributed to many droplets and leads to a blurred expression signal that does not stem solely from the single cell in the droplet but also from the solution that contains it. We used the function inferNonExpressedGenes() from SoupX^63^ to identify a set of 80 ambient RNAs and accounted for these in the downstream analysis.

*Time course differential expression analysis*: to identify genes that show differential expression patterns across time within a given cell type we performed the following analysis. We used the R packages splines and lmtest for our modeling approach. First, we manually combined the Louvain clusters into 26 cell types to generate a more coarse grained cell type annotation for the time course differential expression analysis (Supplementary Fig. 1d). Within each of these groups we modeled gene expression as a binomial response where the likelihood of detection of each gene within each mouse sample was the dependent variable. Therefore, the sample size of the model was the number of mouse samples (*n* = 28) and not the number of cells. To assess significance we performed a likelihood-ratio test between the following two models. For the first model, the independent variables contained an offset for the log-transformed average total UMI count and a natural splines fit of the time course variable with two degrees of freedom. The independent variables of the second model just contained the offset for the log-transformed average total UMI count. The dependent variable of both models was the number of cells with UMI count greater than zero out of all cells for a given cell type and mouse sample. To account for potential false positive signal derived from ambient RNA levels, we calculated cell type marker genes for the 26 cell type annotation using the Seurat FindAllMarkers() function. For all 80 candidate ambient RNAs, we consequently set all regression *p*-values to one in cell types where the gene was not simultaneously a marker gene with an adjusted *p*-value of <0.1 and a positive average log fold change.

*Cell–cell communication analysis*: to identify cell–cell communication networks, we downloaded a list of annotated receptor-ligand pairs^64^. Next, we integrated this information with the cell type marker genes from Supplementary Data 1. Cell–cell communication networks were generated in the following manner. An edge was created between two cell types if these two cell types shared a receptor-ligand pair between them as marker genes.

*Macrophage analysis*: it is not entirely understood whether monocyte-derived macrophages contribute to the development of lung fibrosis. To see if our data reflects published models of monocyte recruitment, we integrated bulk RNAseq data from FACS sorted macrophage populations after bleomycin-induced lung fibrosis^8,65^. This data set contained bulk RNAseq gene expression of tissue-resident alveolar macrophages (TR-AMs), monocyte-derived alveolar macrophages (Mo-AMs), interstitial macrophages (IM), and monocytes (Mono) for both day 14 and day 19 after bleomycin injury, including additional measurements for TR-AMs at day 0. To derive a gene expression signature from the bulk RNAseq data, we used the R package limma^66^. We followed the standard limma workflow^65^ to find genes which are differentially expressed between these four populations. Next, we subset our scRNAseq data set to only clusters expressing known macrophage markers and selected a new set of variable genes. Following this the PCA and UMAPs were recreated for this subset, using 20 PCs and 20 n_neighbors in Seurat’s functions. The macrophages from our data were scored according to their similarity to these bulk-derived signatures using Pearson correlation. For each of the four bulk-derived groups, the log fold changes of the 500 most differentially expressed genes were correlated with the scaled expression values of each macrophage cell in our scRNAseq data. To separate potential monocyte-derived macrophages from interstitial macrophages, we assigned each cell to the category with the higher correlation coefficient as long as the difference was >0.05. Otherwise, the cell was labeled unassigned.

### Processing of the high-resolution epithelial data set

The high-resolution gene expression matrix was generated as specified for the whole-lung data set with the following changes. To lessen the technical bias introduced by ambient RNA, we applied SoupX the pCut parameter set to 0.3 within each sample before merging the count matrices together. The merged expression table was then pre-processed as described in the section “Processing of the whole-lung data set”, with minor alterations. To account for the fact that a certain fraction of the counts was removed, the upper threshold for the number of total UMI counts per cell was set to 3000.

### Analysis of the high-resolution epithelial data set

The computational analysis of the whole-lung data set was performed using a combination of the Seurat^60^ and Scanpy^67^ code. Cell-cycle effects, the percentage of mitochondrial reads, and the total number of UMI counts are often viewed as unwanted sources of variation and were therefore regressed out using the Seurat functions CellCycleScoring() and ScaleData(). Genes which had a variable expression in at least two samples (17038 genes) were used for the principal component analysis. The majority of the cells were airway and alveolar epithelial cells, although non-epithelial cells were also captured. To filter the data further, the cells were clustered and clusters expressing non-epithelial markers were excluded from the data set. The cleaned object was then converted to a.h5ad file for downstream analysis using the python package Scanpy. The aligned bam files were used as input for Velocyto^14^ to derive the counts of unspliced and spliced reads in loom format. Next, the sample-wise loom files were combined, normalized and log transformed using scvelos (https://github.com/theislab/scvelo)^68^ functions normalize_per_cell() and log1p(). After merging the loom information to the exported.h5ad file using scvelos merge() function the object was scaled and the neighbourhood graph constructed with Batch balanced KNN (BBKNN)^69^ to account for the different PCR cycles used in the experiment with neighbors_within_batch set to 15 and n_pcs to 40. Two dimensional visualization and clustering was carried out with the Scanpy functions tl.louvain() at resolution two and tl.umap(). The neuroendocrine cells (NEC) formed a distinct cluster in the UMAP, however, they were only assigned to a single cluster at higher resolutions. To separate them from basal cells we captured the NEC with dbscan using the UMAP coordinates and assigned them as cluster 21. After manual curation of the markers the remaining 20 clusters were combined, leading to thirteen final meta cell types. Relative frequencies were calculated as described for the whole-lung data set. To better visualize the dynamic changes of each cell type over time, values were scaled to a minimum of 0 and a maximum of 1 using numpy’s interp() function for each cell type annotation separately. Smoothed line plots of the scaled frequencies were generated by employing the lmplot() function of the python module seaborn with default parameters.

*Cell-cycle analysis*: the proliferating cells (Louvain cluster 14, Fig. 4d) of the high-resolution data set were subjected to cell type deconvolution analysis. Cell cycle phases (S.Score, G2M.Score) were regressed out using the Seurat ScaleData() function. Next, PCA was calculated using all unique marker genes from Supplementary Data 3 and the Seurat RunPCA() function. UMAP embedding and Louvain clusters were calculated using the first 20 principal components with the Seurat RunUMAP() and FindClusters() functions, respectively. Upon manual curation of the marker genes for the generated embedding, we identified four distinct clusters. Next, the frequency of proliferating cells was calculated by dividing the number of cells in cluster 14, by the number of total cells for each mouse sample.

*PAGA analysis*: to assess the global connectivity topology between the Louvain clusters we applied Partition-based graph abstraction (PAGA)^28^. We applied the tl.paga() function integrated in the Scanpy package to calculate connectivities and used the Louvain clusters as partitions. The weighted edges represent a statistical measure of connectivity between the partitions. Connections with a weight <0.3 were removed.

*Velocity analyses*: to infer future states of individual cells we made use of the spliced and unspliced information. We employed *scvelo*^68^ (https://github.com/theislab/scvelo). The previously normalized and log transformed data was the starting point to calculate first and second order moments for each cell across its nearest neighbors (scvelo.pp.moments(n_pcs = 40, n_neighbors = 15)). Next, the velocities were estimated and the velocity graph constructed using the scvelo.tl.velocity() with the mode set to’stochastic’ and scvelo.tl.velocity_graph() functions. Velocities were visualized on top of the previously calculated UMAP coordinates with the scvelo.tl.velocity_embedding() function. To compute the terminal state likelihood of a subset of cells, the function scvelo.tl.terminal_states() with default parameters was used.

*Trajectory differential expression analysis*: to identify genes showing significantly altered expression along the differentiation trajectories toward the Krt8+ cell state, the following approach was used. The high-resolution data set was restricted to cells from Louvain clusters 2, 10, 11, and 12 for the convergence and AT1 trajectories. The convergence (Louvain clusters 2, 10, 11) and AT1 (Louvain clusters 2, 12) trajectories were analyzed independently. For the convergence and AT1 trajectories we used diffusion map and UMAP as the cellular embeddings, respectively. The dbscan() function from the DBSCAN R package was used to identify outlier cells which were subsequently removed from further analysis. The R package slingshot^70^ was used to infer the pseudotemporal ordering along the trajectory of the cellular embeddings of all remaining cells. Next, the analysis was restricted to genes detected in at least 5% of cells. The R package tradeSeq^71^ was used to identify genes differentially expressed along the trajectories. Despite the fact that *p*-values derived from pseudotemporal analyses are inflated they can be used to prioritize candidate genes. Heatmaps were restricted to genes with Benjamini-Hochberg adjusted *p*-values < 0.05. Gene expression patterns along pseudotemporal trajectories were visualized using local polynomial regression fitting as implemented in the R loess() function with default parameters.

*In silico doublet simulation*: to exclude the potential artefacts derived from cell doublets we performed the following analyses. In silico bulk samples were generated by summing the counts across cells randomly sampled from specific cell clusters or mixtures thereof. More precisely, we randomly selected 600 cells from AT1, AT2, club and Krt8+ ADI cell clusters in silico bulk samples per cell identity. Doublets were generated by randomly selecting 300 cells from the AT2 and AT1 clusters as well as Club and AT1 clusters and subsequently aggregated into in silico samples. This procedure was repeated five times to generate five in silico samples per condition. Counts were normalized using the voom() function of the limma R package and subjected to principal component analysis. Analogous procedure was performed for the club cell analysis.

*Integration of whole-lung and high resolution data sets*: epithelial cells of the whole-lung data set were re-analysed to validate findings derived from the high resolution data set. The principal components were re-calculated on this subset using a new set of variable genes, in order to emphasize changes in the epithelium specifically. Following the procedure described above, UMAP visualization and RNA velocities were generated using Scanpy and scvelo.

### Pathway analysis

To predict the activity of pathways and cellular functions based on the observed gene expression changes, we used the Ingenuity Pathway Analysis platform (IPA, QIAGEN Redwood City, www.qiagen.com/ingenuity) as previously described^24^. The analysis uses a suite of algorithms and tools embedded in IPA for inferring and scoring regulator networks upstream of gene-expression data based on a large-scale causal network derived from the Ingenuity Knowledge Base. We used the upstream regulator tool in IPA to derive pathway *z*-scores across cell type identities by loading the marker gene list fold changes of our single cell louvain clusters (logFC relative to all other cells) for comparison of the indicated cell type identities. The missing values represent cell type signatures that did not have significant overlap with the respective pathways in IPA. The upstream regulator tool in IPA defines an overlap *p* value measuring enrichment of network-regulated genes in the data set, as well as an activation *Z*-score which can be used to find likely regulating molecules based on a statistically significant pattern match of up- and down-regulation, and also to predict the activation state (either activated or inhibited) of a putative regulator. In our analysis we considered pathways/genes with an overlap *p* value of > 7 (log10) that had an activation *Z*-score > 2 as activated and those with an activation *Z*-score < −2 as inhibited.

### Magnetic-activated cell sorting

Cells from whole-lung single cell suspensions were strained using a 40 µm mesh size and red blood cells were eliminated by lysis (RBC lysis buffer, ThermoFisher). For positive epithelial cell selection, cells were stained with CD326-AlexaFluor647 antibody (Biolegend, 118212) for 30 min at 4 °C in the dark, and after washing, incubated with microbeads specific against AlexaFluor647 (Miltenyi Biotec, 130-091-395) for 15 min at 4 °C. MACS LS columns (Miltenyi Biotec, 130-042-401) were prepared according to the manufacturer’s instructions. Cells were applied to the columns and positively-labeled epithelial cells were retained in the column. The flow-through was collected separately for later mesenchymal cell enrichment (negative magnetic-activated cell sorting (MACS) selection) and kept on ice. Epithelial cells were eluted from the LS columns and used for either Dropseq runs. Mesenchymal cells from the flow-through were further enriched by negative depletion of CD31+ (Invitrogen, 17-0311-82), CD45+ (Biolegend, 103112), Lyve1+ (Invitrogen, 50-0443-82), Ter119+ (Biolegend, 116218), and CD326+ cells (Biolegend, 118212). After antibody staining, 100 µl per 10 million cells of MACS dead cell removal beads (Miltenyi Biotec, 130-090-101) were added and incubated according to the product’s accompanying protocols. Depletion of undesired cell types was achieved by the use of microbeads specific for APC (Miltenyi Biotec, 130-090-855), which ensured magnetic retention of these cells. Likewise to epithelial cells, negatively-selected mesenchymal cells were applied to the Dropseq workflow.

### Flow cytometry

Isolated total lung cell suspensions were used to detect and quantify cell populations by flow cytometry. After depletion of red blood cells by red blood cell lysis buffer (Invitrogen, ThermoFisher), cell suspensions were stained with anti-mouse CD45-PE-Vio770 (Miltenyi Biotec, 130-110-661), CD326-BV421 (Biolegend, 118225), Krt8/TROMA-I (DSHB-Developmental Studies Hybridoma Bank at the University of Iowa), and αvβ6-specific monoclonal antibody 6.3G9 (Itgb6-3G9; kindly provided by Prof. Dr. Dean Sheppard, available through Biogen Idec, USA). Cells were stained for surface markers in the dark at 4 °C for 20 min, followed by cell fixation and permeabilization (Fix & Perm, Life Technologies, GAS004) for intracellular staining of Krt8. Epithelial cells were selected using the CD45-negative fraction of the cell isolate that stained positively for CD326. Within the epithelial cell gate, Krt8+, Itgb6+, or Krt8+/Itgb6+ cells were identified and quantified by their geometric mean fluorescence signal intensity. For exclusion of non-specific antibody binding and autofluorescence signal, fluorescence minus one (FMO) controls were included in the measurement. All stainings were performed per 1,000,000 cells in the following dilutions: CD326 (1:500), CD45 (1:20), Krt8 (1:35), Itgb6 (1:1000). Data was acquired in a BD LSRII flow cytometer (Becton Dickinson, Heidelberg, Germany) and analyzed by mean fluorescence intensity (MFI) using the FlowJo software (TreeStart Inc., Ashland, OR, USA). Negative thresholds for gating were set according to isotype-labeled and unstained controls.

### Precision cut lung slices (PCLS)

Precision cut lung slices were generated as previously described^72^. Briefly, using a syringe pump, the mouse lungs were filled via a tracheal cannula with 2% (w/v) warm, low gelling temperature melting point agarose (Sigma Aldrich, A9414) in sterile DMEM/Ham’s F12 cultivation medium (Gibco, 12634010), supplemented with 100 U/ml penicillin, 100 µg/ml streptomycin, and 2.5 µg/ml amphotericin B (Sigma Aldrich, A2942). Afterwards, the lungs were removed and transferred on ice in cultivation medium for 10 min to allow for gelling of the agarose. Each lung lobe was separated and cut with a vibratome (Hyrax V55; Zeiss, Jena, Germany) in 300 µm thick sections. The PCLS were immediately fixed in −20 °C-cold methanol for 20 min and subsequently stained for immunofluorescence microscopy.

### Immunofluorescence microscopy of PCLS and analysis

Methanol-fixed PCLS were stained and imaged as previously described^73^. Shortly, primary antibodies were diluted in 1% bovine serum albumin (BSA, Sigma Aldrich, 84503) in PBS (1:100), incubated for 16 h at 4 °C and subsequently washed three times with PBS for 5 min each. Secondary antibodies were diluted in 1% bovine serum albumin in PBS (1:200), incubated for 4 h at room temperature and subsequently washed three times with PBS for 5 min each. Primary antibodies were: rat anti-Krt8/TROMA-I (1:200; DSHB-Developmental Studies Hybridoma Bank at the University of Iowa), rabbit anti-pro-SPC (1:200; Millipore, AB3786), goat anti-Pdpn (1:200; R&D Systems, AF3244). Cell nuclei were stained with DAPI (40,6-diamidino-2-phenylindole, Sigma-Aldrich, 1:2,000). Confocal high-resolution 3D imaging of the PCLS was accomplished by placing the PCLS into a glass-bottomed 35 mm CellView cell culture dish (Greiner BioOne, 627870) as a wet chamber. Images of PCLS were acquired as z-stacks using an inverted microscope stand with an LSM 710 (Zeiss) confocal module operated in multitrack mode using the following objectives: Plan-Apochromat W 40×/1.0 M27 and Plan-Apochromat W 63×/1.3 M27. The automated microscopy system was driven by ZEN2009 (Zeiss) software, version 5.5. The acquired confocal fluorescent z-stacks were surface rendered in Imaris 9.3 software (Bitplane) and its statistical analysis tool (MeasurementPro) was used for 3D cell shape analysis using morphometric parameter sphericity as a readout (a value of 1 corresponds to a perfect sphere).

### Immunofluorescence microscopy

After euthanasia, mouse lungs were immediately inflated with 4% paraformaldehyde. For frozen OCT embedding, tissue was fixed for 1 h at room temperature. Thin lung sections (7 µm) were cut on a cryostat. Sections were incubated with 0.1% sodium borohydride (PBS) to reduce background fluorescence, followed by blocking in PBS plus 1% bovine serum albumin, 5% non-immune horse serum (UCSF Cell Culture Facility), 0.1% Triton X-100 (Carl Roth, 3051.3) and 0.02% sodium azide (Sigma Aldrich, S2002). Slides were then incubated in primary antibodies overnight at 4 °C followed by secondary antibody incubation at 1:1,000 dilutions at room temperature for >1 h. Slides were counterstained with 1 µM DAPI for 5 min at room temperature and mounted using Prolong Gold (Life Technologies, P36930). The following antibodies were used: rabbit anti-pro-SPC (1:2,500; Millipore, AB3786), and rat anti-Krt8 (0.9 µg/ml; TROMA-I (Krt8); University of Iowa Hybridoma Bank). Slides were imaged using a Leica Microscope (DM6B-Z; Leica Biosystems) or Axivision Imager M1 (Carl Zeiss AG).

For formalin-fixed, paraffin-embedded (FFPE) lung tissue, sections were cut at 3.5 µm, followed by deparaffinization, rehydration, and antigen retrieval by pressure-cooking (30 s at 125 °C and 10 s at 90 °C) in citrate buffer (10 mM, pH 6.0). After blocking for 1 h at room temperature with 5% bovine serum albumin, lung sections were incubated in primary antibodies overnight at 4 °C, followed by secondary antibody (1:250) incubation for 2 h at room temperature. The following primary (1) and secondary (2) antibodies were used: (1) rat anti-Krt8 (170 µg/ml; University of Iowa Hybridoma Bank, 1:200), rabbit anti-pro-SPC (1:200; Millipore, AB3786), goat anti-Pdpn (1:200; R&D Systems, AF3244), rabbit anti-SPC (1:150; Sigma-Aldrich, HPA010928), mouse anti-alphaSMA (1:1,000, Sigma-Aldrich, A5228), rabbit anti-Areg (1:50; LSBIO, LS-B13911), rabbit anti-Hbegf (1:200; Bioss Antibodies, bs-3576R), rabbit anti-Ki67 (1:200; Abcam, ab16667), mouse anti-CC10 (1:200; Santa Cruz, sc-365992), rabbit anti-Cst3 (1:100; Abcam, ab109508), rabbit anti-Yap (1:500; Abcam, ab205270), rabbit anti-pSmad2 (Ser465/467) (1:1000; Cell Signaling, 3101), rabbit anti-Krt17 (1:200; Sigma, HPA000452); (2) donkey anti-rabbit AlexaFluor568 (Invitrogen, A10042), donkey anti-rat AlexaFluor488 (Invitrogen, A21208), donkey anti-goat AlexaFluor647 (Invitrogen, A21447), goat anti-mouse AlexaFluor647 (Invitrogen, A21236). Images were acquired with an LSM 710 microscope (Zeiss).

### Microscopic image analysis–quantification

The fluorescence intensity of Krt8 expression in selected regions of immunofluorescence microscopy images was measured excluding airways using FIJI (ImageJ, v.1.8.0)^74^. For quantification of Krt8 expression in the human FFPE sections, and likewise, for the Hbegf and Areg quantification in the mouse sections, the mean overall fluorescence intensities were measured. For quantification of cell proliferation, cells were stained with Ki67 and Krt8 and counted manually for Ki67 positive cells.

### Lineage tracing experiments

SPC-CreERT2 (Sftpc^tm1(cre/ERT2,rtTA)Hap^) mice were crossed with Gt(ROSA)26Sor^tm4(ACTB-tdTomato,-EGFP)Luo^ mice. Sox2-CreERT2 mice were crossed with Ai14-tdTomato (Gt(ROSA)26Sor^tm14(CAG-tdTomato)Hze^). Four doses (SPC-CreERT2) or three doses (Sox2-CreERT2) of 0.25 mg/g body weight tamoxifen in 50 μl corn oil. A chase period of >21 days was used to ensure the absence of residual tamoxifen before injury. Bleomycin (1.5 U/kg) was delivered to mouse lungs via oral aspiration in 40 µL sterile PBS. Lungs were harvested at 10 days or 14 days following injury.

### Edu labeling and Sox2-CreERT2

Sox2-CreERT2/tdTomato mice were labeled with tamoxifen dissolved in corn oil (3 doses, 250 mg/kg) followed by two weeks of chase before injuring with bleomycin dissolved in 1X PBS (2.1U/kg). Proliferating cells were labeled with 5-Ethynyl-2′-deoxyuridine (Edu) every other day starting two days after bleomycin injury (50 mg/kg dissolved in 1X PBS, intraperitoneal injection). Lungs were harvested twenty days post bleomycin injury, embedded in optimal cutting temperature compound (OCT) and stained for Edu using Click-iT Edu Imaging kit (ThermoFisher, c10086). Images were quantified by counting the total number of proliferating AEC1s (Edu+/RAGE1+) in two-three lobes/mouse (*n* = 2 mice). Each dot represents one large region (>600 DAPI+ cells each) from one lobe.

### Uninjured labeling

Spc-CreERT2 or Sox2-CreERT2 mice were labeled with tamoxifen (4 or 3 doses at 250 mg/kg, respectively). Lungs were harvested at least one week post last dose of tamoxifen and OCT embedded sections were stained for KRT8. At least one lobe/mouse was quantified for Spc-CreERT2 mice (*n* = 3). Each dot represents quantification of a large region from one lobe of a mouse. We found no labeling of alveolar cells in Sox2-CreERT2 mice.

### Hypoxia/Hyperoxia+InfA infection model

Wild-type or bi-transgenic Sftpc^CreERT2^; Rosa26R^mTmG^ mice were exposed to 12% (hypoxia), 21% (room air) or 100% (hyperoxia) oxygen between postnatal days 0–4^75^. All mice were then exposed to room air until they were 8 weeks old. Sftpc^CreERT2^; Rosa26R^mTmG^ mice were administered tamoxifen (Sigma Aldrich, T5648) (0.25 g/kg) or corn oil vehicle by single daily injections for four consecutive days^76^. On the seventh day, the mice were infected with influenza A virus (HKx31, H3N2) and lungs were harvested on post-infection day 14. Lungs were inflation fixed overnight in 10% neutral buffered formalin, embedded in paraffin, sectioned and stained with antibodies against pro-SPC (Seven Hills Bioreagents, Cincinnati, OH);

T1alpha (1:100; Syrian Hamster, clone 8.1.1, DSHB-Developmental Studies Hybridoma Bank at the University of Iowa) and Krt8/TROMA-I (DSHB-Developmental Studies Hybridoma Bank at the University of Iowa); Sections were incubated with fluorescently labeled secondary antibody and stained with 4’, 6-diamidino-2-phenylindole (DAPI). Slides were visualized with a Nikon E-800 fluorescence microscope (Nikon Instruments, Microvideo Instruments, Avon, MA). Images were captured with a SPOT-RT digital camera (Diagnostic Instruments, Sterling Heights, MI).

### pmATII isolation and culture

Primary mouse ATII cells (pmATII) were isolated from 8 to 10 week-old, pathogen-free, female C57BL6/N mice (Charles River Laboratories, SUuzfeld, Germany) as previously described^59,77^. Briefly, lungs were filled with dispase (Corning, New York, NY, USA) and low-gelling temperature agarose (Sigma Aldrich, Saint Louis, MO, USA) before mincing and filtering through 100-, 20-, and 10-μm nylon meshes (Sefar, Heiden, Switzerland). Fibroblasts were depleted by adherence on non-coated plastic plates. Macrophages and white blood cells were depleted using CD45-specific magnetic beads (Miltenyi Biotec, Bergisch Gladbach, Germany), and endothelial cells with CD31-specific magnetic beads, respectively. Cell depletion was performed according to the manufacturer’s instructions. pmATII cells were resuspended in DMEM containing 10% FCS (PAA Laboratories, Pasching, Austria), 2 mM glutamine, 1% penicillin/streptomycin (both Life Technologies, Carlsbad, CA), 3.6 mg/ml glucose (Applichem GmbH, Darmstadt, Germany) and 10 mM HEPES (PAA Laboratories), and cultured for 24 h to allow for cell attachment. The medium was changed to medium containing 7.5 µM ICG-001 (Biomol) or the respective DMSO control, refreshed at day 3. Cells were cultured up to 5 days.

### Western blotting

Cells were washed with PBS (PAA Laboratories), lysed in T-PER lysis buffer (Thermo Fisher Scientific, Waltham, MA), supplemented with proteinase inhibitor cocktail tablets (Roche, Germany). Protein concentration was quantified using the Pierce BCA Protein Assay Kit (Pierce, Thermo Fisher Scientific) according to the manufacturer’s instructions. In all, 10 µg of protein lysates were separated on SDS-polyacrylamide gel and transferred to nitrocellulose membranes. Membranes were blocked with 5% non-fat dried milk solution in TRIS-buffered saline containing 0.01% (v/v) Tween (TBS-T) (Applichem, Darmstadt, Germany) for 1 h and incubated with primary T1α (R&D Systems) or Krt8/TROMA-I (DSHB-Developmental Studies Hybridoma Bank at the University of Iowa) antibody at 4 °C overnight. Next, blots were incubated for 1 h at RT with secondary, HRP-conjugated, antibodies (GE-Healthcare), or HRP-conjugated anti-β-actin antibody (Sigma-Aldrich) prior to visualization of the bands using chemiluminescence reagents (Pierce ECL, Thermo Scientific, Ulm, Germany). Blots were recorded with the ChemiDocTMXRS+ system and analyzed using the Image Lab 6.0.1 software (Biorad, Munich, Germany).

### Reporting summary

Further information on research design is available in the Nature Research Reporting Summary linked to this article.

## Supplementary information

Supplementary Information

Reporting Summary

Description of Additional Supplementary Files

Supplementary Data 1

Supplementary Data 2

Supplementary Data 3

Supplementary Data 4

Supplementary Data 5

Supplementary Data 6

## Data Availability

Bulk and scRNA-seq data are available via the Gene Expression Omnibus with the accession code GSE141259. Additionally, results can be explored using our interactive webtool at https://theislab.github.io/LungInjuryRegeneration.

## Code availability

Code to reproduce the analyses described in this manuscript can be accessed via: https://github.com/theislab/2019_Strunz.
