# Supplementary material for: Alveolar regeneration through a Krt8+ transitional stem cell state that persists in human lung fibrosis
Source: Nat Commun. 2020 Jul 16;11:3559. doi: 10.1038/s41467-020-17358-3 (PMC7366678; doi:10.1038/s41467-020-17358-3)
Supplement: Supplementary file 1 — Supplementary Information [file 41467_2020_17358_MOESM1_ESM.pdf]

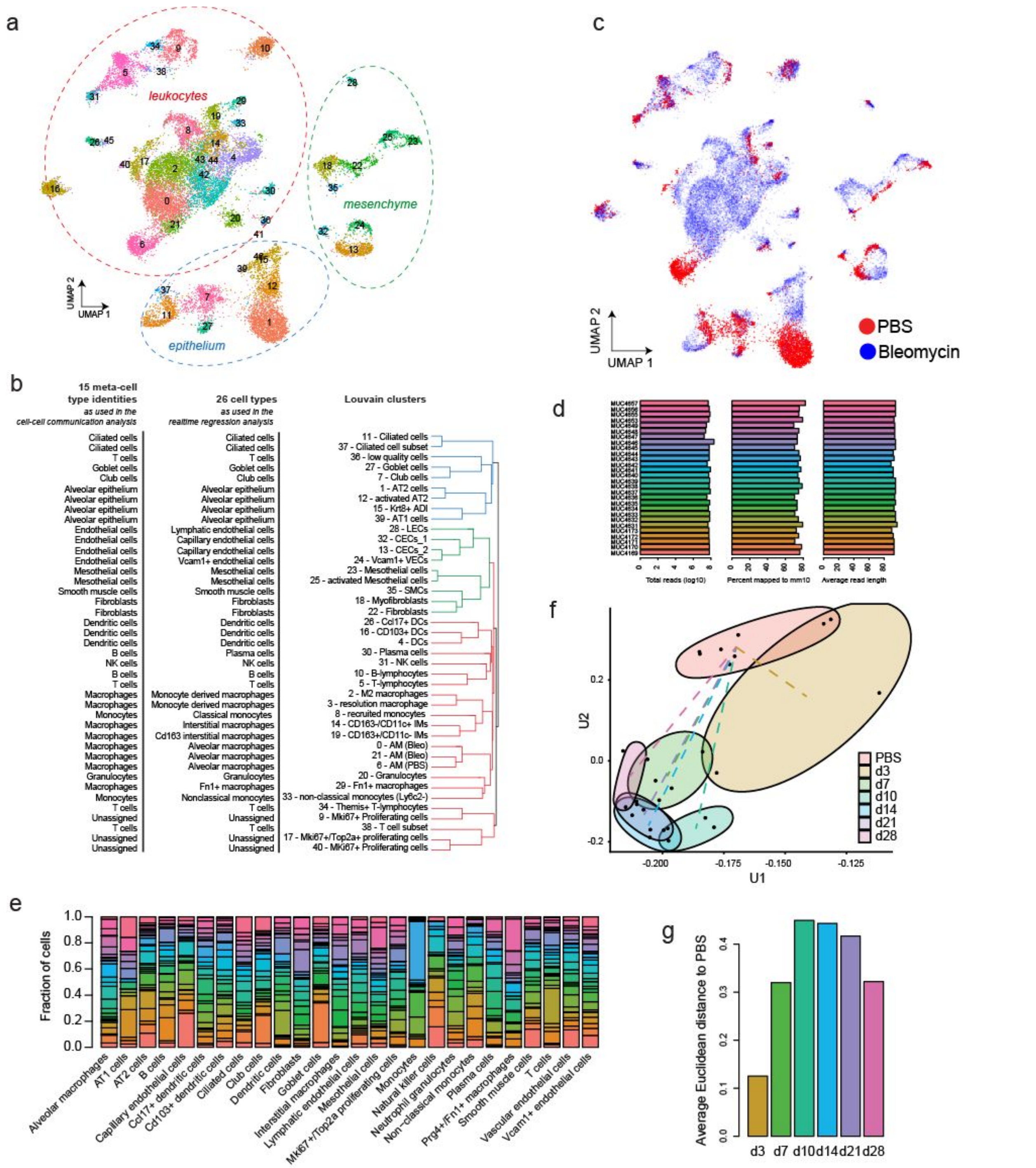

Supplementary Figure 1. Good technical agreement of whole lung single cell transcriptomes of 28 individual mice. (a) UMAP embedding colored by Louvain clusters demonstrates separation of cells into major lineages. (b) Unsupervised hierarchical clustering of the Louvain clusters recapitulates known hierarchical cell type topology. UMAP embeddings show good overlap between treatment conditions (c). (d) Alignment summary statistics are comparable across mouse samples. (e) Bar plot shows high overlap of mouse samples across cell types. (f) Scatter

plot depicts coordinates from singular value decomposition of the arcsine square root transformation of the relative cell type frequencies. Dashed lines connect the mean coordinates between PBS and all other time points. Ellipses are colored by time point and encapsulate samples from the same time point. (g) Barplot displays mean Euclidean distance derived from the embedding in (f) between PBS and all other time points.

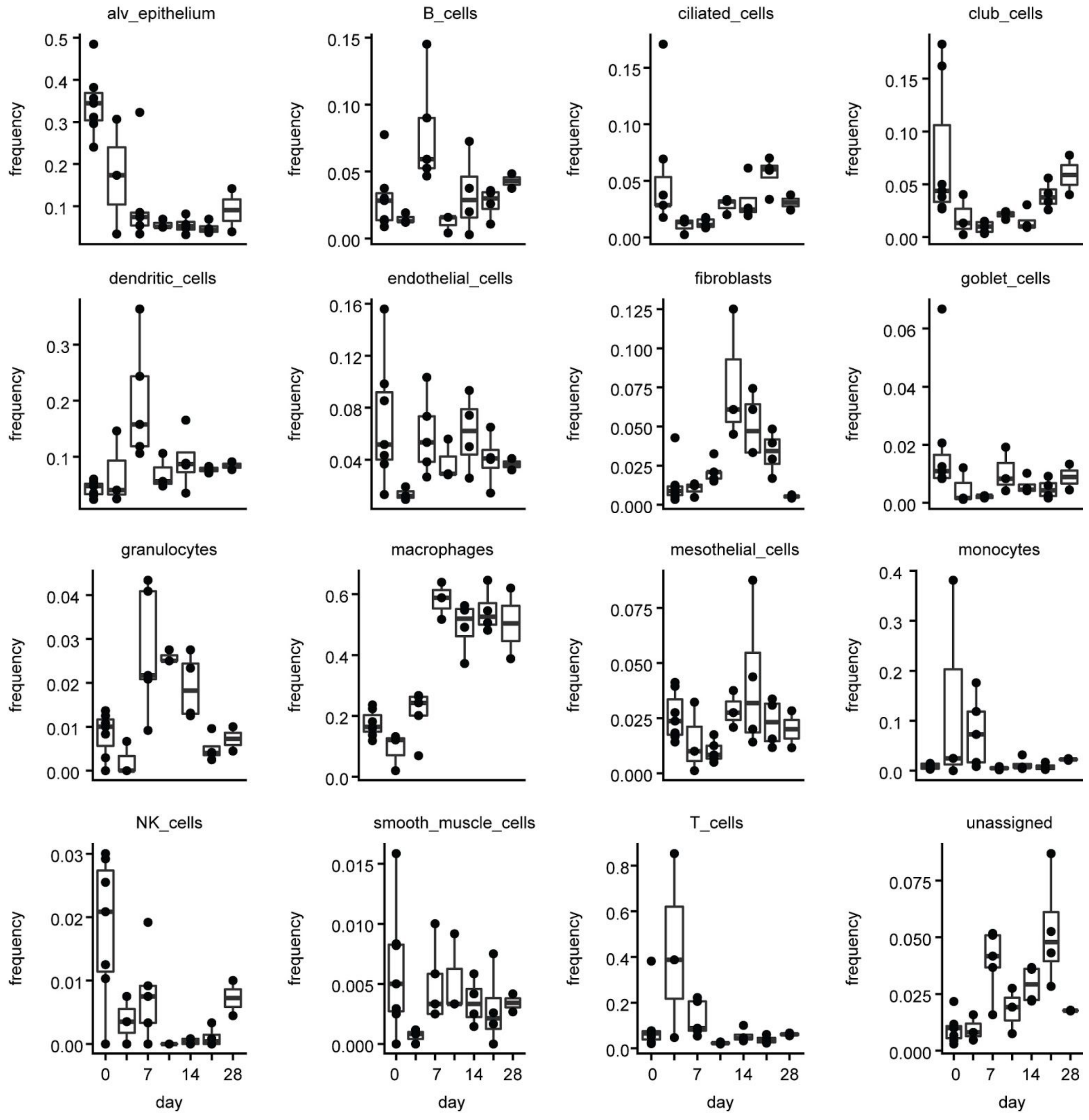

Supplementary Figure 2. Barplots display the relative frequencies (y-axis) of 16 meta-cell types across time points (x-axis) across 28 mouse replicates. Each dot represents one mouse sample. The boxes represent the interquartile range, the horizontal line in the box is the median, and the whiskers represent 1.5 times the interquartile range.

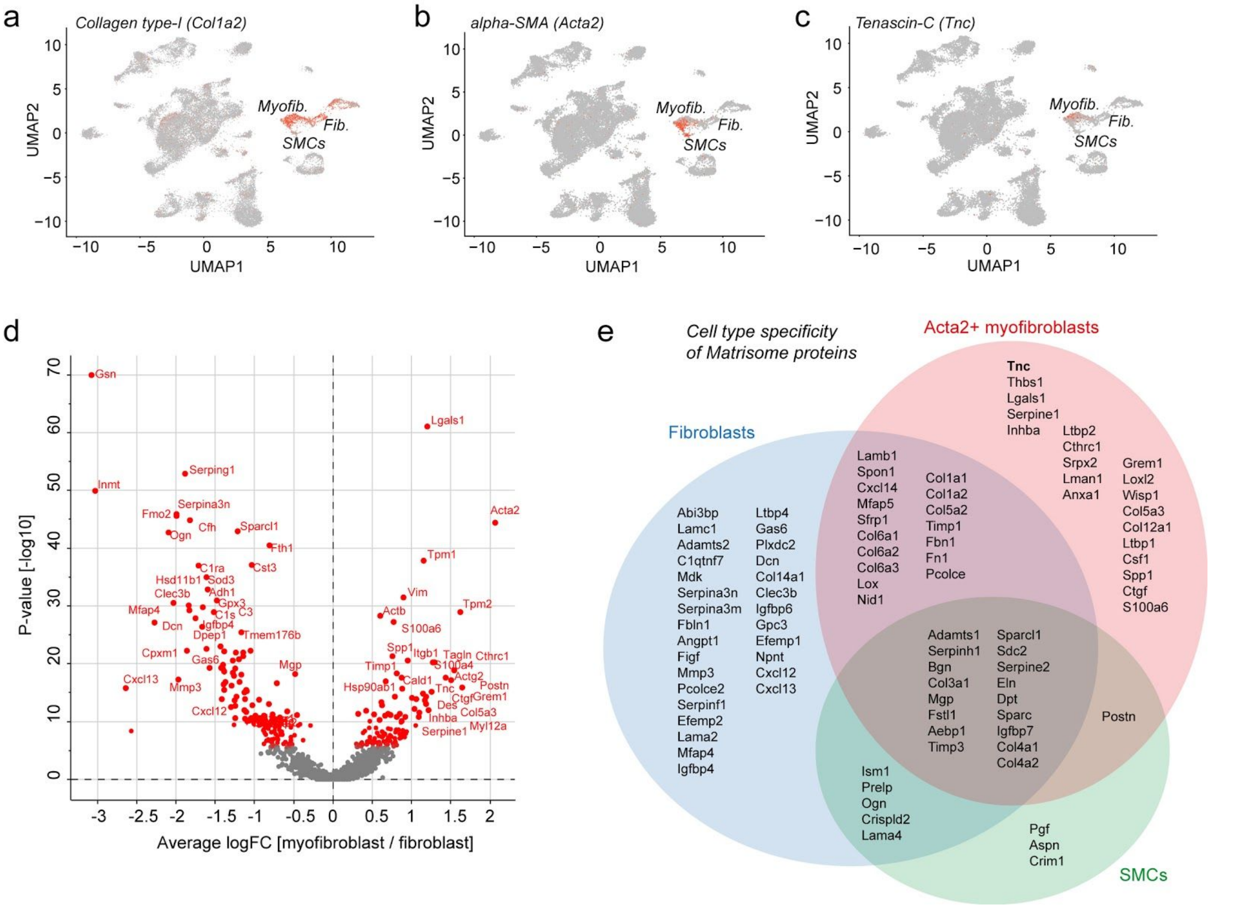

Supplementary Figure 3. Transient appearance of the myofibroblast cell state upon lung injury. (a-c) Relative expression levels of Col1a2 (a), Acta2 (b), and Tnc (c) are shown on the UMAP embedding. (d) The volcano plot shows differential gene expression between myofibroblasts (right side) and fibroblasts (left side). (e) Single cell analysis was used to derive the myofibroblast specific ECM components in comparison to other fibroblasts and smooth muscle cells.

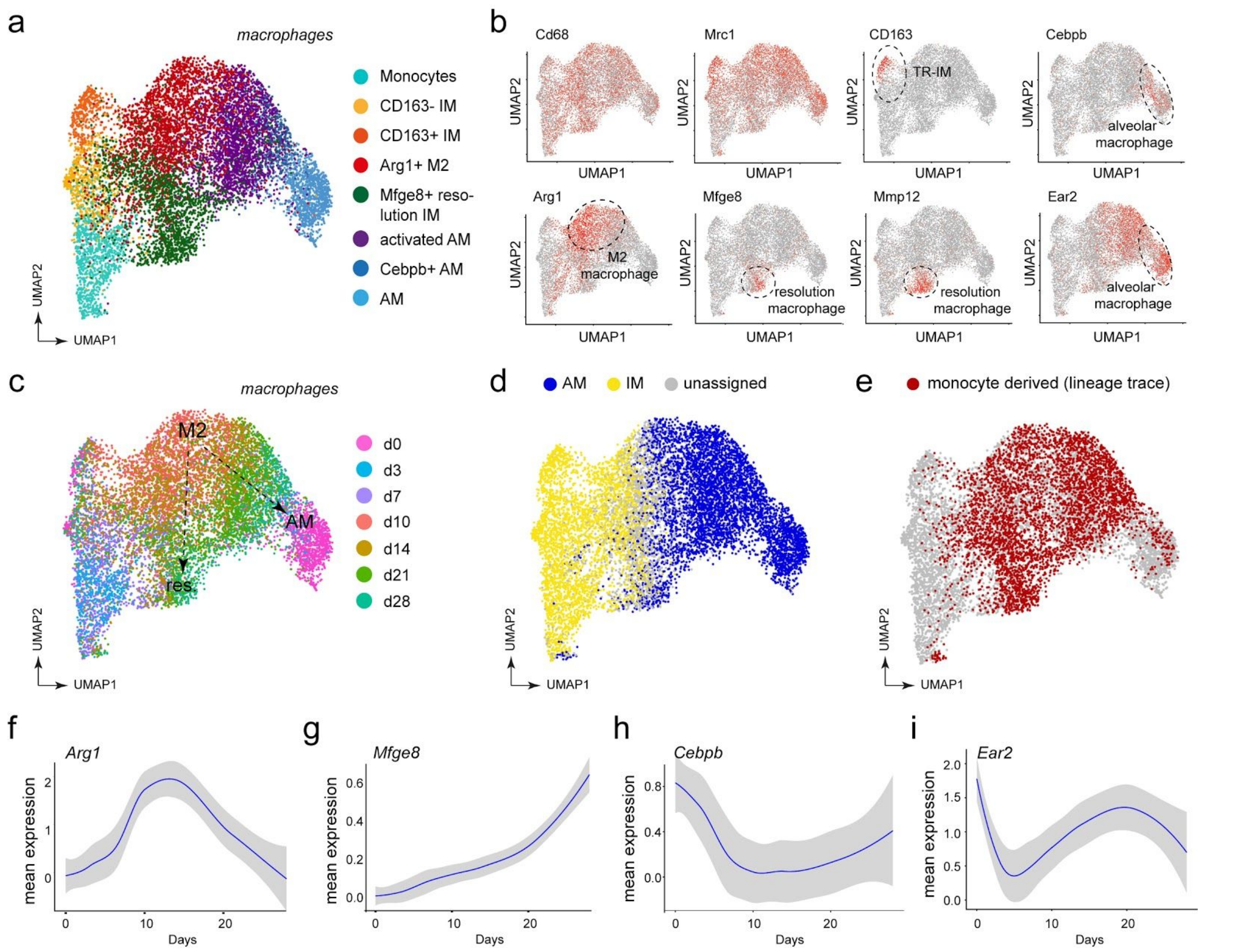

Supplementary Figure 4. Dynamics of macrophage states in lung tissue regeneration. (a, c) UMAP embedding of 10379 cells that express known macrophage markers is colored by (a) cluster identity and (c) time points. Following cells along the time course after reaching the peak of inflammation at day 10 and 14, two potential trajectories can be discerned. (b) Several macrophage populations can be identified. These clusters uniformly express the macrophage marker Cd68 and Mrc1 while also showing distinct expression of certain genes. (d) Previously published gene signatures from bulk RNA experiments were used to reveal potential origins of macrophage cells. In this data set, FACS-sorting allowed to differentiate between tissue-resident alveolar (AM), interstitial (IM) and monocyte-derived macrophage populations[9]. Similarity score of each cell is calculated as correlation to differentially expressed genes and corresponding log fold changes in the three sorted populations. Cells are assigned to either AM or IM category, if the difference in scores for either category is higher than 0.05. Alveolar macrophages in our data set indeed show the highest score on the tissue-resident AM. (e) Potentially monocyte-derived cells based on scoring (at threshold of 0.1). There is a separation in the potentially monocyte-derived cells, which concurs with the real-time trajectories in (c). (f-i) The line plots show the smoothed expression mean over time for the indicated genes within all macrophage subsets with a confidence interval of 0.95 (grey shades) across the 28 mouse replicates.

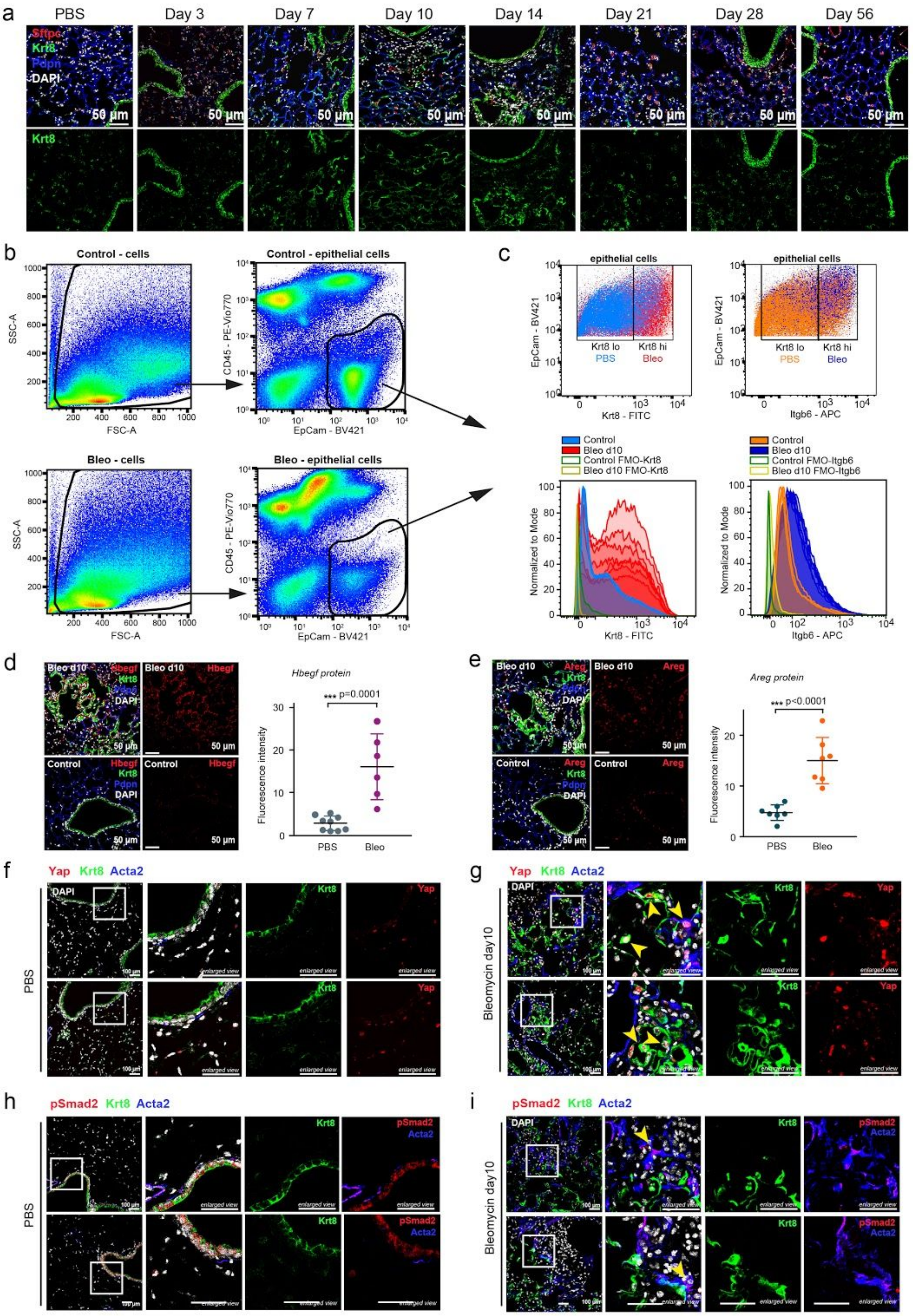
a
PBS
Day 3
Day 7
Day 10
Day 14
Day 21
Day 28
Day 56
Sftpc
Krt8
Pdpn
DAPI
50 µm
Krt8
b
Control - cells
Control - epithelial cells
Bleo - cells
Bleo - epithelial cells
SSC-A
FSC-A
CD45 - PE-Vio770
EpCam - BV421
c
epithelial cells
Krt8 lo
Krt8 hi
PBS
Bleo
EpCam - BV421
Krt8 - FITC
Itgb6 - APC
Control
Bleo d10
Control FMO-Krt8
Bleo d10 FMO-Krt8
Control FMO-Itgb6
Bleo d10 FMO-Itgb6
Normalized to Mode
d
Bleo d10
Hbegf
Control
Hbegf protein
*** p=0.0001
Fluorescence intensity
PBS
Bleo
e
Areg
Areg protein
*** p<0.0001
f
Yap Krt8 Acta2
DAPI
enlarged view
100 µm
g
Bleomycin day10
h
pSmad2 Krt8 Acta2
i

Supplementary Figure 5. Protein validation of the alveolar Krt8+ ADI signature. (a) Immunostaining of Krt8 (green) at the indicated time points after bleomycin injury. FFPE tissue sections were co-stained with the AT2 marker Sftpc (red), and the AT1 marker Pdpn (blue). Nuclei were labeled using DAPI (white). Scale bar = 50 microns; representative images from n=4 lungs/timepoint. (b) Gating strategy for the analysis of CD45-/Epcam+ epithelial cells. (c) The scatter plots and histograms show increased expression of Krt8 and Itgb6 at day 10 after bleomycin in Epcam+ epithelial cells. Highest expression of Itgb6 was observed on Krt8 high cells. Fluorescence-minus-one (FMO) controls were used for both the Krt8 and Itgb6 quantification. (d) Increased Hbegf (red) expression in bleomycin treated lung tissue, showing partial overlap with Krt8 (green) signal. Quantification of the mean fluorescence signal intensities confirmed increased Hbegf expression (unpaired t-test, one-sided, *** $p = 0.0001$, mean measure with SD). Sections were co-stained with Pdpn (blue); scale bar = 50 microns. Sections assessed in PBS n=9, in Bleo n=6. (e) Immunostainings of Areg (red) and Krt8 (green) expression in the lung, co-stained with Pdpn (blue) and quantified by mean fluorescence intensity. Unpaired t-test, one-sided, *** $p < 0.0001$, mean measure with SD. Scale bar = 50 microns. Sections assessed in PBS n=7, in Bleo n=7. (f-i) Tissue sections from either controls (f, h) or bleomycin day 10 (g, i) were stained with the indicated antibodies to assess activity of the Yap/Taz pathway (f, g) and the TGF-beta signaling by pSMAD nuclear translocation (h, i); representative images from n=2 lungs/condition.

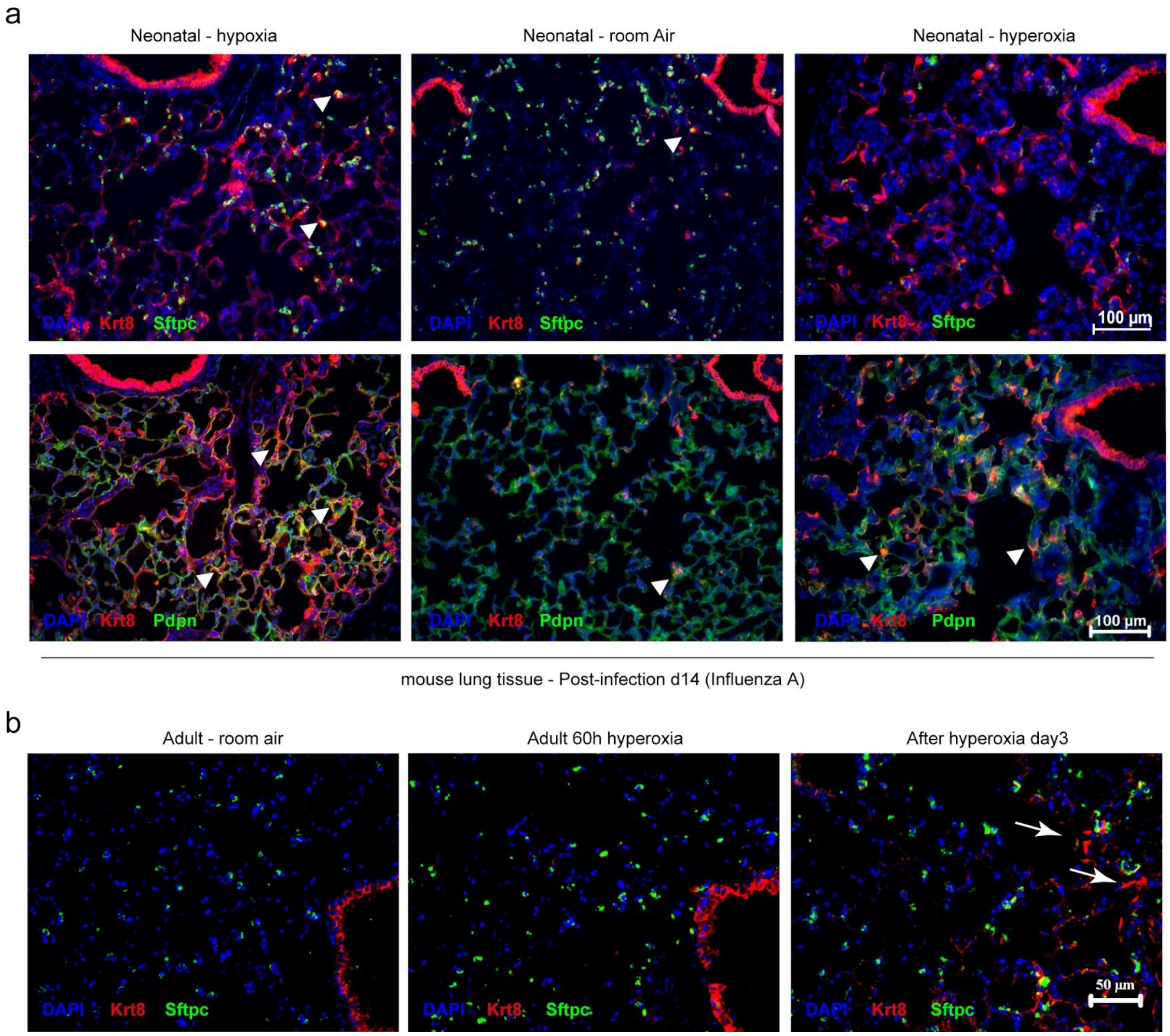

Supplementary Figure 6. Appearance of Krt8+ ADI cells in two alternative mouse injury models. (a) An aberrant oxygen environment at birth alters alveolar injury and repair following influenza A virus infection. Lungs of infected mice that were previously exposed to the indicated neonatal conditions were stained for Krt8 (red) and Sftpc (green). Scale bar = 100 microns. (b) A sixty-hour exposure of adult mice to hyperoxia leads to the emergence of Krt8+ cells in the alveolar space. Mice were sacrificed three days after the exposure period terminated. Lung tissue was stained for Krt8 (red) and Sftpc (green). Scale bar = 50 microns.

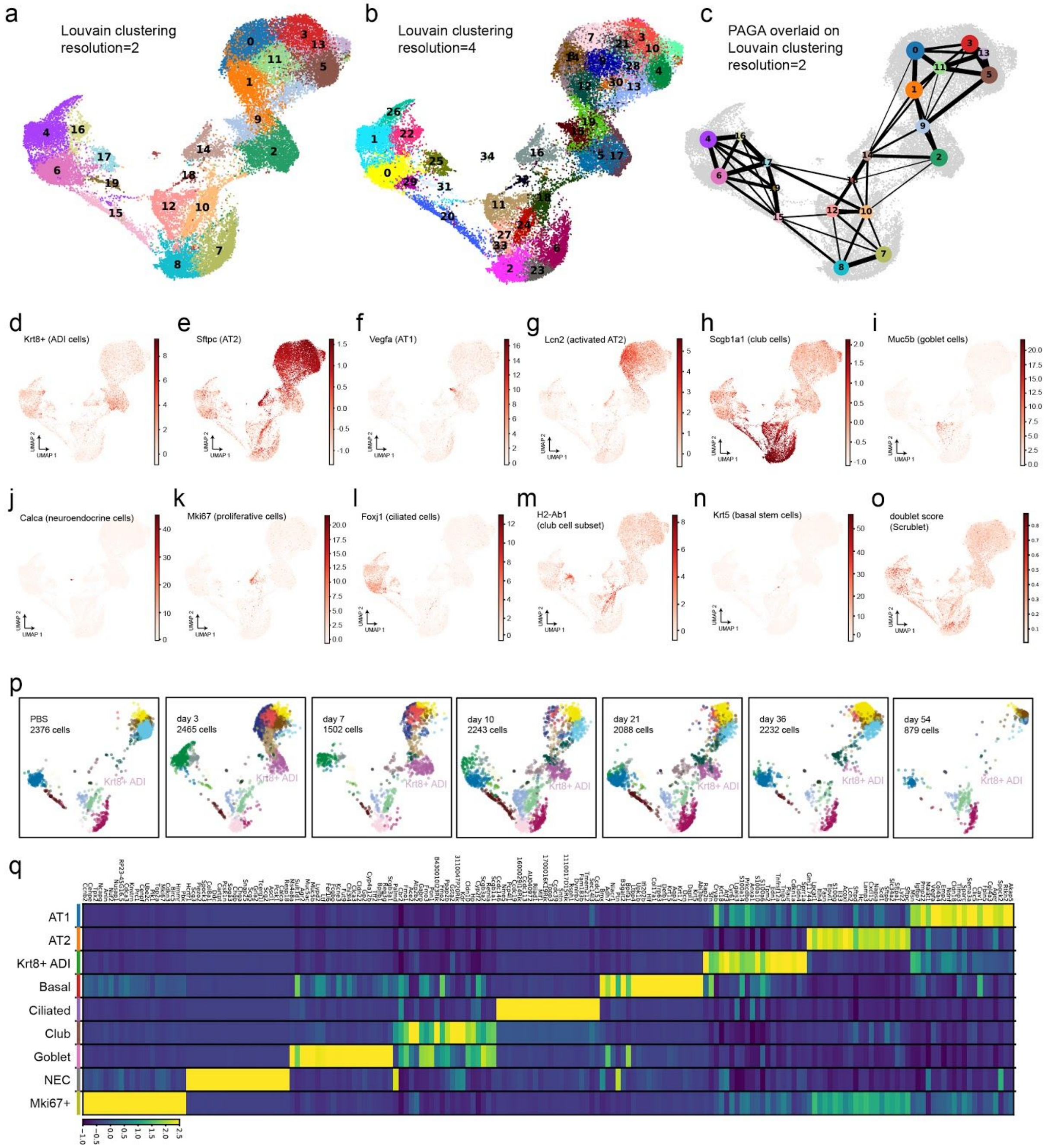

Supplementary Figure 7. Feature plots of selected marker genes for epithelial cell types and states. (a,b) UMAP embeddings showing the Louvain clustering as calculated with the resolution parameter=2 (a) and resolution parameter=4 (b). (c) PAGA graph representation of the data overlaid onto the UMAP representation with respective Louvain clustering, calculated with resolution parameter=2. (d-n) UMAP embeddings display distinct expression patterns for selected epithelial cell type marker genes: (d) Krt8 (ADI), (e) Sftpc (AT2 cells), (f)Vegfa (AT1 cells), (g) Lcn2 (activated AT2 cells), (h) Scgb1a1 (club cells), (i) Muc5b (goblet cells), (j) Calca (neuroendocrine cells), (k) Mki67 (proliferative cells), (l) Foxj1 (ciliated cells), (m) H2-Ab1 (club cell subset), (n) Krt5 (basal stem cells). Red colors indicate higher expression levels, (o) doublet score calculated with the doublet detection algorithm Scrublet. (p) UMAP shows epithelial cells at the indicated time points after injury color coded by their

respective louvain cluster identity (Fig. 5). Note the massive increase of Krt8+ ADI relative frequency during inflammation and fibrogenesis. (q) Heatmap shows the average expression levels for the top 20 genes with lowest adjusted p value of each cell type.

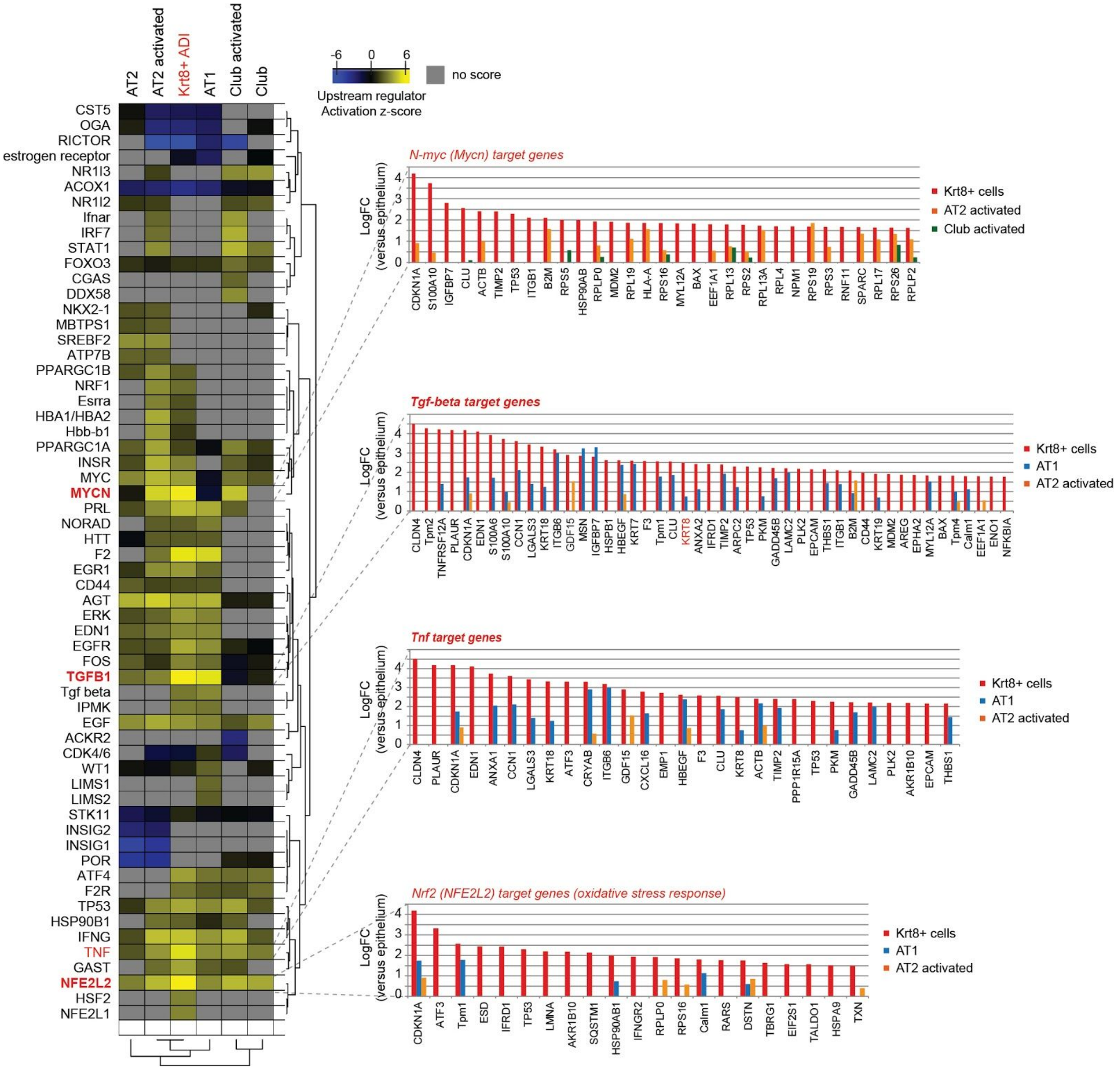

Supplementary Figure 8. Gene programs with increased activity in Krt8+ ADI. Ingenuity upstream regulator analysis was used to score the activity of upstream regulators within the signatures of the indicated cell states. The activation z-scores were grouped by hierarchical clustering using their Pearson correlation. Bar graphs show target genes sorted by highest expression in Krt8+ cells relative to all other cells.

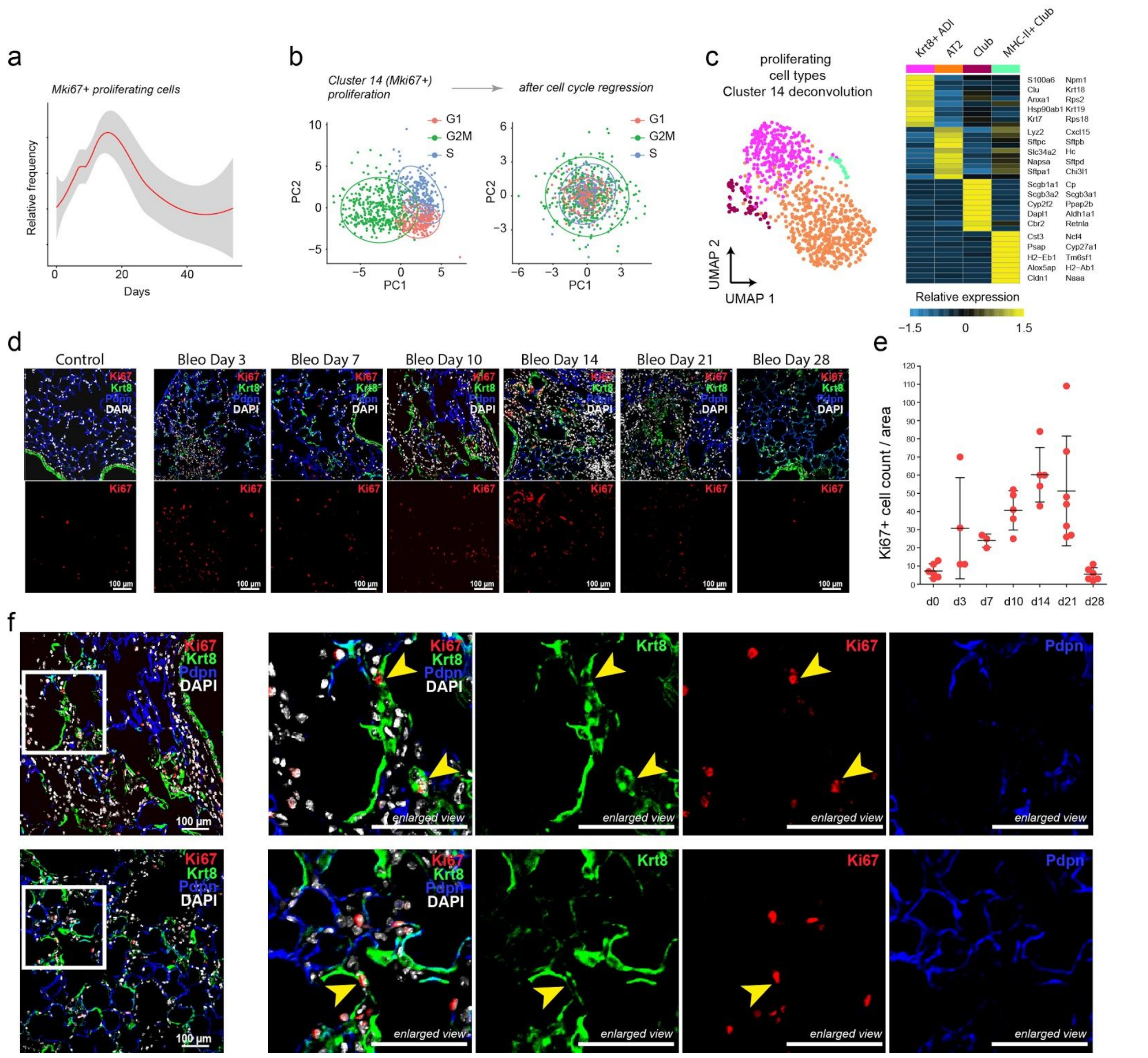

Supplementary Figure 9. Cell cycle analysis shows no massive expansion of rare pre-existing Krt8+ ADI cells. (a) The line plot shows the smoothed relative frequency of Mki67+ proliferating cells over time. Grey colors indicate 95% confidence interval of fit across the 36 mouse replicates. (b) The scatter plots show cells from proliferating cell cluster 14 before and after cell cycle regression, colored by inferred cell cycle phase. Regression removes cell cycle effects from principal component data manifold. Re-analysis of cell cycle corrected expression deconvolves cell type identities of proliferating cells. (c) UMAP of cell cycle corrected cluster 14 cells visualizes four distinct clusters, which contain Krt8+ ADI, AT2, club, and MHC-II+ club cells. Heatmap shows the average expression levels of selected marker genes. (d) Immunofluorescence stainings of control versus bleomycin treated lung sections (day 3, 7, 10, 14, 21, 28). Sections were stained for Krt8 (green), Ki67 (red), Pdpn (blue), and DAPI (white). Scale bar indicates 100 microns. (e) Ki67+ cells were quantified from the micrographs by counting Ki67+ cells in each ROI/field of view [mean with SD, n(d0)=6, n(d3)=4, n(d7)=3, n(d10)=5, n(d14)=5, n(d21)=7, n(d28)=6]. (f) Immunostaining as in (e) on day 10 post bleomycin injured lungs with enlarged views on proliferative Krt8+ ADI cells (Ki67+/Krt8+), highlighted with yellow arrowheads. Scale bar indicates 100 microns, n(mice)=2.

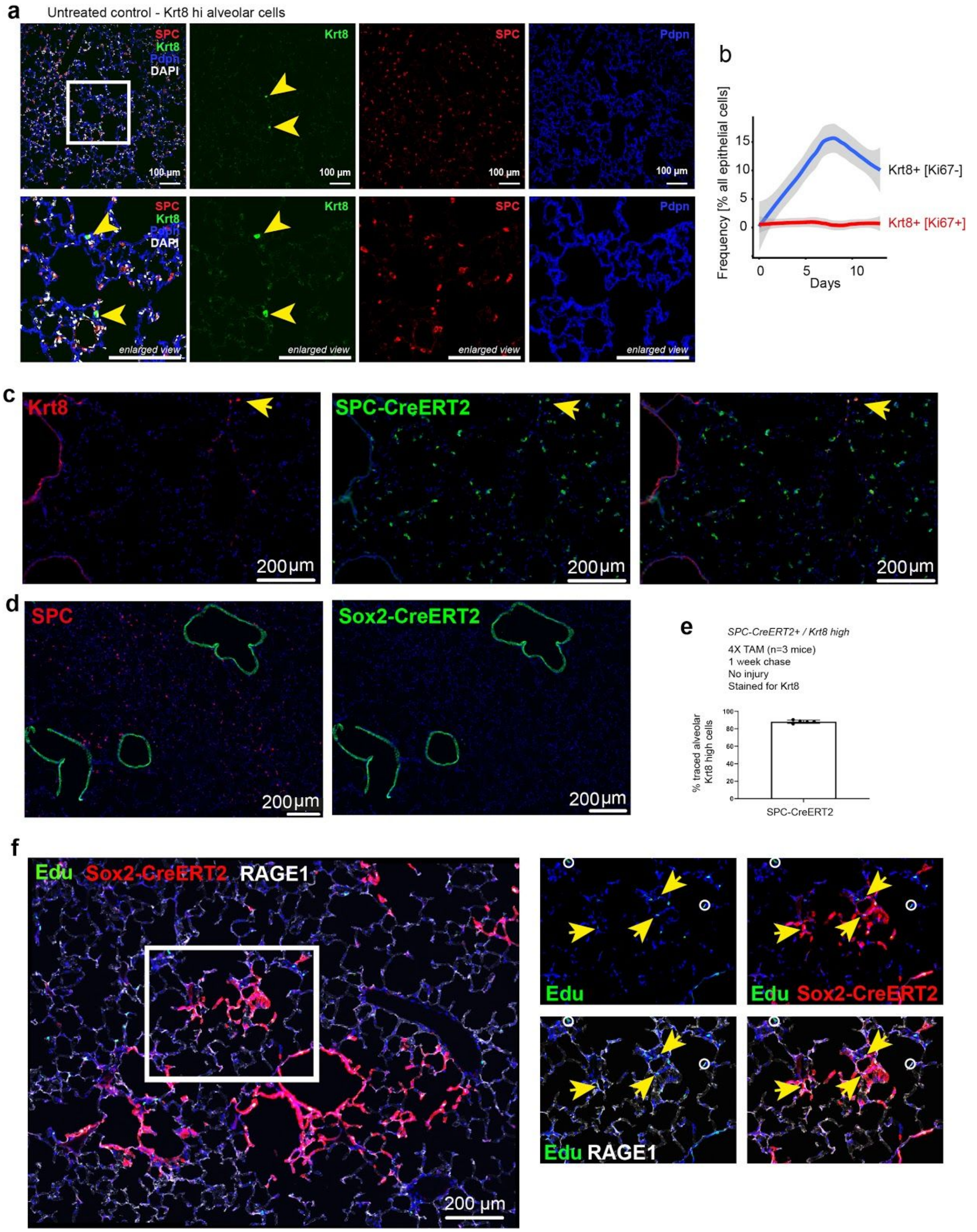

Supplementary Figure 10. Rare Krt8 high alveolar cells in healthy lung parenchyma. (a) Fluorescent immunostainings and confocal imaging of lung sections from untreated control lungs. Nuclei (DAPI) are colored in white, Krt8 appears in green, Sftpc (AT2 cells) in red, and Pdpn (AT1 cells) in blue. The scale bar indicates 100 microns. (b) Line plots show smoothed relative frequency of cells with a Krt8+ ADI signature stratified in Ki67+ (red

line) and Ki67- bins (blue line). Note the massive expansion of Krt8+ ADI over time without spiking numbers of Ki67+ cells preceding this. This indicates that most Krt8+ ADI are generated by differentiation of stem cells rather than expansion of pre-existing Krt8 high cells from the baseline. Grey colors indicate 95% confidence interval of a fit across the mouse replicates. (c, d) SPC-CreERT2 mice (c, n=3) or Sox2-CreERT2 mice (d) were labeled for 1 week using 4x tamoxifen (TAM) injections. Cryosections were stained for (c) Krt8 or SPC (d) and the lineage label as shown. (e) Quantification of SPC-CreERT2 traced cells with high expression of Krt8 in alveolar areas shows that most pre-existing rare Krt8 high alveolar cells are AT2 cell derived; n(mice)=3 Each data point represents quantification from one large region (n=6; each at least 2.5sqmm area). Error bars show standard deviation. (f) Cryosection of an Edu-labeled lung after bleomycin-injury in the Sox2-CreERT2 mice; the four enlargements as indicated by the white box show that proliferating Edu+/RAGE1+ cells have overlapping signal with the Sox2 lineage label (yellow arrows), indicating that Sox2 lineage-derived cells can give rise to RAGE1+ AT1 cells; untraced double positive Edu+/RAGE1+ cells are highlighted by a white circle. Experiment includes n=2 mice; at least 3 lobes/mouse were analyzed with similar results.

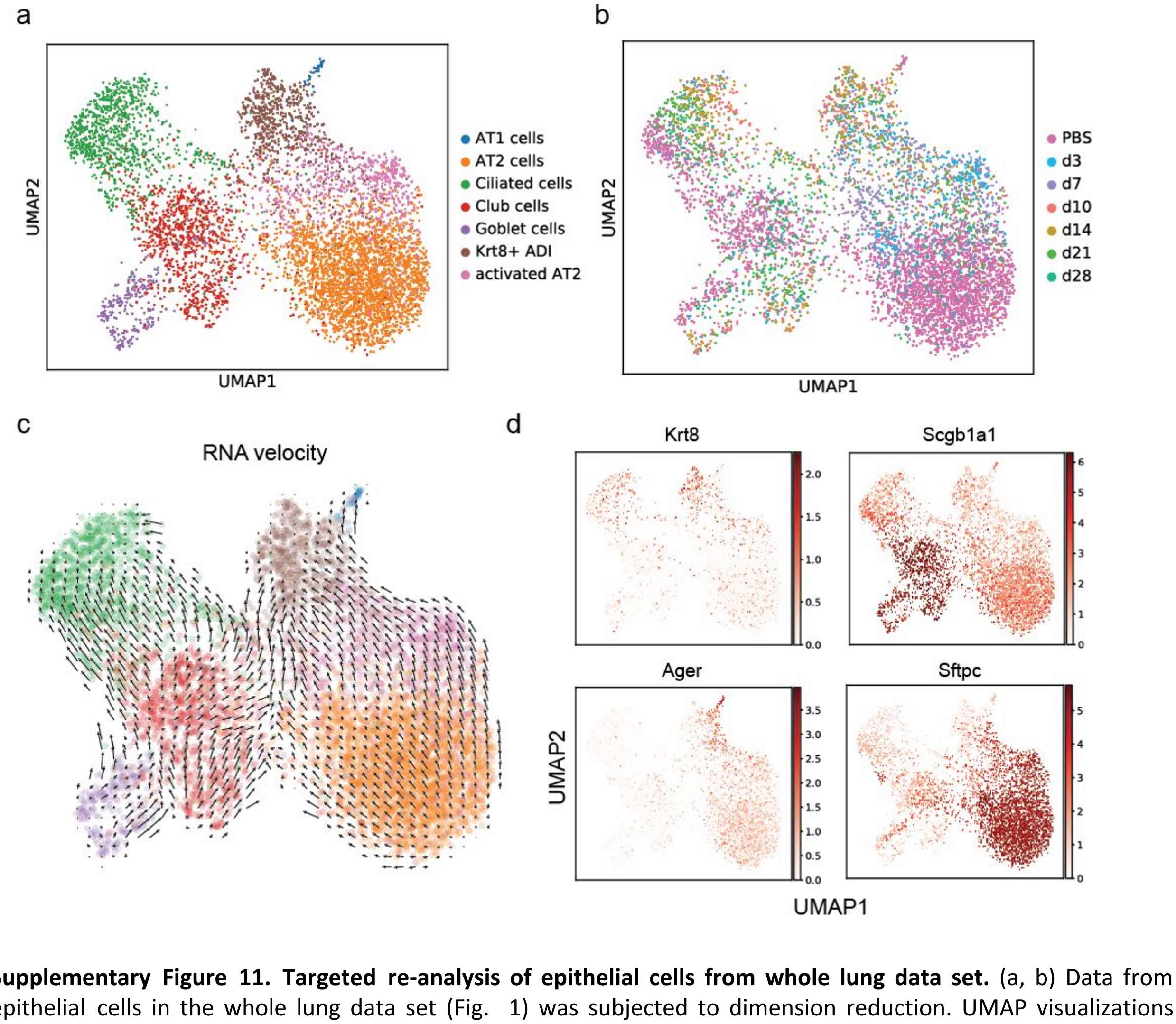

Supplementary Figure 11. Targeted re-analysis of epithelial cells from whole lung data set. (a, b) Data from epithelial cells in the whole lung data set (Fig. 1) was subjected to dimension reduction. UMAP visualizations colored by cell type (a) and time point (b) illustrate injury-specific cells connecting major cell types. (c) RNA

velocities predict both AT2 and airway cell derived Krt8+ ADI. (d) Expression of select marker genes is colored on top of the UMAP embedding. Grey and red colors correspond to low and high values, respectively.

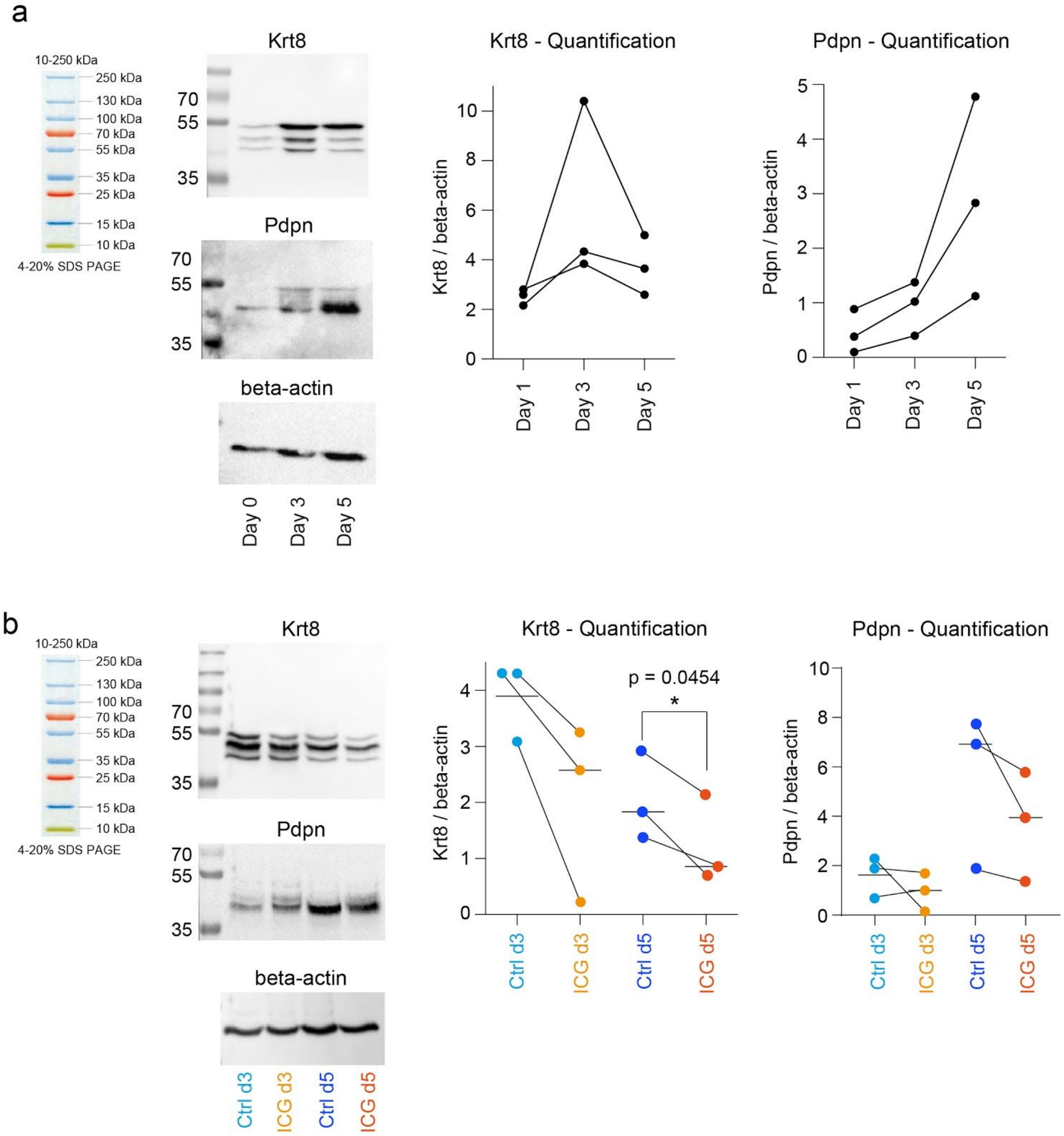

Supplementary Figure 12: AT1 cell differentiation involves Wnt/β-catenin/TCF-mediated transcription. (a) Representative Western blot for the detection of Krt8 and Pdpn in MACS-negatively selected pmAT2 cells plated on plastic dishes; proteins were analyzed and quantified at day 1, day 3, and day 5, demonstrating that during in vitro differentiation the expression of Krt8 (peak expression at day 3) and Pdpn are increased over time (n of blots = 3). All gels/blots were processed in parallel. (b) Representative Western blot for likewise selected pmAT2 cells, treated with WNT inhibitor ICG-001 (start of inhibition at day 1). Inhibition was stopped at day 3 and day 5, respectively, and lysates loaded for Western blot analysis. WNT inhibition induced a reduction in both Krt8 and Pdpn levels compared to untreated control samples (n of blots = 3, data shown with mean). Quantification of the Krt8 protein at day 5 revealed a significant decrease of protein levels upon WNT inhibition (paired t-test, two-sided, p = 0.0454). All gels/blots were processed in parallel.

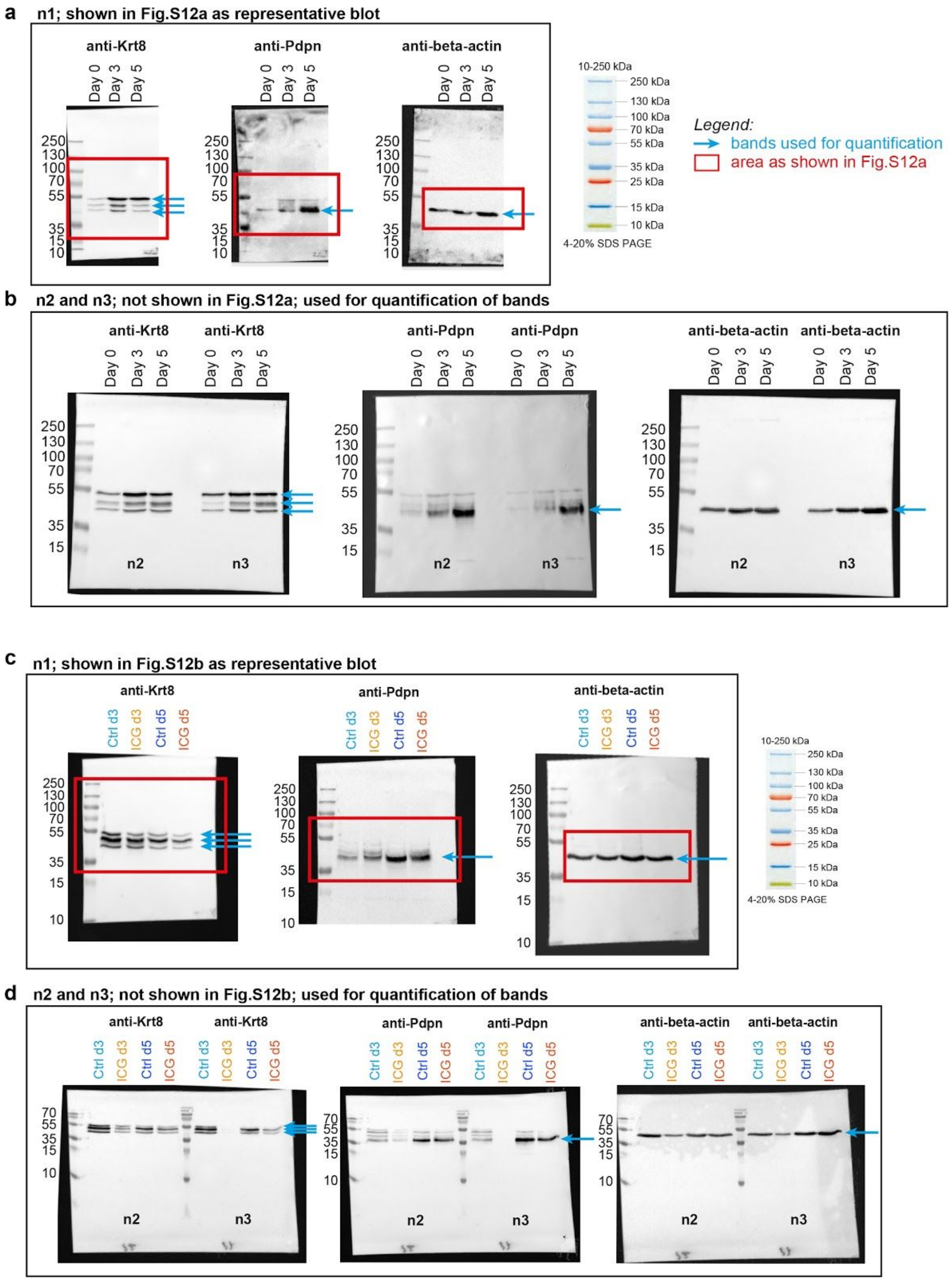

Supplementary Figure 13. Full-scan sizes of all western blots from supplementary figure 12. A total of n=3 samples were used for all blots. (a) The first sample used as a representative blot. (b) The second and third sample blots which were included in the blot quantification. (c) The first sample used as a representative blot for the inhibition experiment. (d) The second and third sample blots which were included in the blot quantification of the inhibition experiment.
