# Supplementary material for: Alveolar regeneration through a Krt8+ transitional stem cell state that persists in human lung fibrosis
Source: Nat Commun. 2020 Jul 16;11:3559. doi: 10.1038/s41467-020-17358-3 (PMC7366678; doi:10.1038/s41467-020-17358-3)
Supplement: Supplementary file 2 — Reporting Summary [file 41467_2020_17358_MOESM2_ESM.pdf]

nature research

Corresponding author(s): Dr. Herbert Schiller, Prof. Dr. Fabian Theis

Last updated by author(s): May 25, 2020

Reporting Summary

Nature Research wishes to improve the reproducibility of the work that we publish. This form provides structure for consistency and transparency in reporting. For further information on Nature Research policies, see Authors & Referees and the Editorial Policy Checklist .

Statistics

For all statistical analyses, confirm that the following items are present in the figure legend, table legend, main text, or Methods section.

n/a	Confirmed	
☐	☒	The exact sample size (*n*) for each experimental group/condition, given as a discrete number and unit of measurement
☐	☒	A statement on whether measurements were taken from distinct samples or whether the same sample was measured repeatedly
☐	☒	The statistical test(s) used AND whether they are one- or two-sided<br>*Only common tests should be described solely by name; describe more complex techniques in the Methods section.*
☐	☒	A description of all covariates tested
☐	☒	A description of any assumptions or corrections, such as tests of normality and adjustment for multiple comparisons
☐	☒	A full description of the statistical parameters including central tendency (e.g. means) or other basic estimates (e.g. regression coefficient) AND variation (e.g. standard deviation) or associated estimates of uncertainty (e.g. confidence intervals)
☐	☒	For null hypothesis testing, the test statistic (e.g. *F*, *t*, *r*) with confidence intervals, effect sizes, degrees of freedom and *P* value noted<br>*Give P values as exact values whenever suitable.*
☒	☐	For Bayesian analysis, information on the choice of priors and Markov chain Monte Carlo settings
☒	☐	For hierarchical and complex designs, identification of the appropriate level for tests and full reporting of outcomes
☐	☒	Estimates of effect sizes (e.g. Cohen's *d*, Pearson's *r*), indicating how they were calculated

Our web collection on statistics for biologists contains articles on many of the points above.

Software and code

Policy information about availability of computer code

Data collection	Single-cell RNA sequencing reads processing: Dropseq pipeline (v.2.0); Imaging: ZEN2009 v.5.5, Imaris v.9.3; ImageJ/Fiji v.1.8.0
Data analysis	Analysis was performed using the Seurat (version 2.0) R package and the Scanpy (version 1.2.0) Python package. Analysis code is available at: https://github.com/theislab/2019_Strunz

For manuscripts utilizing custom algorithms or software that are central to the research but not yet described in published literature, software must be made available to editors/reviewers. We strongly encourage code deposition in a community repository (e.g. GitHub). See the Nature Research guidelines for submitting code & software for further information.

Data

Policy information about availability of data

All manuscripts must include a data availability statement. This statement should provide the following information, where applicable:

- Accession codes, unique identifiers, or web links for publicly available datasets
- A list of figures that have associated raw data
- A description of any restrictions on data availability

The data are available at Gene Expression Omnibus with accession identifier GSE141259 as well as at our interactive webtool https://theislab.github.io/LungInjuryRegeneration.

Field-specific reporting

Please select the one below that is the best fit for your research. If you are not sure, read the appropriate sections before making your selection.

Life sciences study design

All studies must disclose on these points even when the disclosure is negative.

Sample size	The number of mice used for the studies was mainly based on the historical use of specific time points of the bleomycin mouse model. Derived from the first anaylsis, we decided to go with a daily sampling strategy. Most of the differences that we observed were statistically significant, demonstrating the suitability of the sample size, which were:<br>Whole lung single cell RNAseq experiment (n=28 mice, k=29297)<br>Epithelial high resolution single cell RNAseq experiment (n=36 mice, k=34575)
Data exclusions	For the single-cell RNAseq data, cells were filtered for quality control. Otherwise, no data were excluded.
Replication	The results reported in this manuscript were reproducible in independent experiments as stated in the legends.
Randomization	Mice were randomized to different treatment and timepoint groups.
Blinding	Investigators were not blinded to group allocation.

Reporting for specific materials, systems and methods

We require information from authors about some types of materials, experimental systems and methods used in many studies. Here, indicate whether each material, system or method listed is relevant to your study. If you are not sure if a list item applies to your research, read the appropriate section before selecting a response.

Materials & experimental systems

n/a	Involved in the study	
☐	☒	Antibodies
☒	☐	Eukaryotic cell lines
☒	☐	Palaeontology
☐	☒	Animals and other organisms
☐	☒	Human research participants
☒	☐	Clinical data

Methods

n/a	Involved in the study	
☒	☐	ChIP-seq
☐	☒	Flow cytometry
☒	☐	MRI-based neuroimaging

Antibodies

Antibodies used	MACS: anti mouse CD326-AlexaFluor647 (Biolegend, 118212), anti mouse CD31-APC (Invitrogen, 17-0311-82), anti mouse CD45-APC (Biolegend, 103112), anti mouse Lyve1-APC (Invitrogen, 50-0443-82), anti mouse Ter119-APC (Biolegend, 116218);<br>Flow Cytometry: anti-mouse CD45-PE-Vio770 (Miltenyi Biotec, 130-110-661), anti mouse CD326-BV421 (Biolegend, 118225), anti mouse Krt8 (DSHB, TROMA-I), and anti mouse αvβ6 (Itgb6-3G9; kindly provided by Prof. Dr. Dean Sheppard)<br>Microscopy: rat anti-Krt8 (DSHB, TROMA-I), rabbit anti-pro-SPC (Millipore, AB3786), goat anti-Pdpn (R&D Systems, AF3244), rabbit anti-SPC (Sigma-Aldrich, HPA010928), mouse anti-alphaSMA (Sigma-Aldrich, A5228), rabbit anti-Areg (LSBIO, LS-B13911), rabbit anti-Hbegf (Bioss Antibodies, bs-3576R), rabbit anti-Ki67 (Abcam, ab16667), mouse anti-CC10 (Santa Cruz, sc-365992), rabbit anti-Cst3 (Abcam, ab109508), rabbit anti-Yap (Abcam, ab205270), rabbit anti-pSmad2 (Ser465/467) (Cell Signaling, 3101), rabbit anti-Krt17 (Sigma, HPA000452), donkey anti-rabbit AlexaFluor568 (Invitrogen, A10042), donkey anti-rat AlexaFluor488 (Invitrogen, A21208), donkey anti-goat AlexaFluor647 (Invitrogen, A21447), goat anti-mouse AlexaFluor647 (Invitrogen, A21236)
Validation	All antibodies have been validated by the manufacturer and by multiple citations for reactivity against mouse or human. For FACS, compensation beads and FMO controls have been additionally used.<br>For immunostaining: all antibodies used in mouse or human tissue have been validated by the manufacturers (cf. antibodies above) and by multiple citations for mouse or human reactivity and for use in immunofluorescence staining. Where not tested for previous projects, negative controls were performed.

Eukaryotic cell lines

Policy information about cell lines

Cell line source(s)	*State the source of each cell line used.*
Authentication	*Describe the authentication procedures for each cell line used OR declare that none of the cell lines used were authenticated.*
Mycoplasma contamination	*Confirm that all cell lines tested negative for mycoplasma contamination OR describe the results of the testing for mycoplasma contamination OR declare that the cell lines were not tested for mycoplasma contamination.*

Commonly misidentified lines (See ICLAC register) | *Name any commonly misidentified cell lines used in the study and provide a rationale for their use.*

Palaeontology

Specimen provenance | *Provide provenance information for specimens and describe permits that were obtained for the work (including the name of the issuing authority, the date of issue, and any identifying information).*

Specimen deposition | *Indicate where the specimens have been deposited to permit free access by other researchers.*

Dating methods | *If new dates are provided, describe how they were obtained (e.g. collection, storage, sample pretreatment and measurement), where they were obtained (i.e. lab name), the calibration program and the protocol for quality assurance OR state that no new dates are provided.*

☐ Tick this box to confirm that the raw and calibrated dates are available in the paper or in Supplementary Information.

Animals and other organisms

Policy information about studies involving animals; ARRIVE guidelines recommended for reporting animal research

Laboratory animals

For bleomycin experiments: C57BL/6J mice (all female) were purchased from Charles River Germany and maintained at the appropriate biosafety level at constant temperature and humidity with a 12 hour light cycle. At the age of 9-12 weeks, mice were instilled with bleomycin or PBS.
For lineage tracing experiments:
Sox2 with bleomycin (all female mice): SPC-CreERT2 (Sftpctm1(cre/ERT2,rtTA)Hap) mice were crossed with Gt(ROSA)26Sortm4 (ACTB-tdTomato,-EGFP)Luo mice. Sox2-CreERT2 mice were crossed with Ai14-tdTomato (Gt(ROSA)26Sortm14(CAG-tdTomato) Hze).
Hypoxia/hyperoxia treatment (all female mice): Wild-type or bi-transgenic SftpcCreERT2; Rosa26RmTmG mice were exposed to 12% (hypoxia), 21% (room air) or 100% (hyperoxia) oxygen between postnatal days 0-4. All mice were then exposed to room air until they were 8 weeks old.

Wild animals | The study did not involve wild animals.

Field-collected samples | The study did not involve samples collected from the field.

Ethics oversight | All animal experiments had been approved by the local veterinary authorities (the Government for the administrative region of Upper Bavaria (Regierung Oberbayern, Germany) for Bleomycin experiments

Note that full information on the approval of the study protocol must also be provided in the manuscript.

Human research participants

Policy information about studies involving human research participants

Population characteristics | Human lung material was received from the CPC bioArchive and from the Institute of Pathology as indicated in the method section. As researchers we were not given any detailed patient information except for medically-confirmed diagnosis, disease status and the interal patient-ID.

Recruitment | Human lung FFPE sections were distributed by the CPC bioArchive at the Comprehensive Pneumology Center, Munich. Experts in human biomaterial and data distribution from the CPC bioArchive selected suitable patients and provided ready-to-use FFPE sections.

Ethics oversight | Local ethics committee of the Ludwig Maximilians University, Munich, Germany (333-10)

Note that full information on the approval of the study protocol must also be provided in the manuscript.

Clinical data

Policy information about clinical studies

All manuscripts should comply with the ICMJEguidelines for publication of clinical research and a completedCONSORT checklist must be included with all submissions.

Clinical trial registration | *Provide the trial registration number from ClinicalTrials.gov or an equivalent agency.*

Study protocol | *Note where the full trial protocol can be accessed OR if not available, explain why.*

Data collection | *Describe the settings and locales of data collection, noting the time periods of recruitment and data collection.*

Outcomes | *Describe how you pre-defined primary and secondary outcome measures and how you assessed these measures.*

ChIP-seq

Data deposition

- [ ] Confirm that both raw and final processed data have been deposited in a public database such as GEO.
- [ ] Confirm that you have deposited or provided access to graph files (e.g. BED files) for the called peaks.

Data access links<br>*May remain private before publication.*	*For "Initial submission" or "Revised version" documents, provide reviewer access links. For your "Final submission" document, provide a link to the deposited data.*
Files in database submission	*Provide a list of all files available in the database submission.*
Genome browser session<br>(e.g. UCSC)	*Provide a link to an anonymized genome browser session for "Initial submission" and "Revised version" documents only, to enable peer review. Write "no longer applicable" for "Final submission" documents.*

Methodology

Replicates	*Describe the experimental replicates, specifying number, type and replicate agreement.*
Sequencing depth	*Describe the sequencing depth for each experiment, providing the total number of reads, uniquely mapped reads, length of reads and whether they were paired- or single-end.*
Antibodies	*Describe the antibodies used for the ChIP-seq experiments; as applicable, provide supplier name, catalog number, clone name, and lot number.*
Peak calling parameters	*Specify the command line program and parameters used for read mapping and peak calling, including the ChIP, control and index files used.*
Data quality	*Describe the methods used to ensure data quality in full detail, including how many peaks are at FDR 5% and above 5-fold enrichment.*
Software	*Describe the software used to collect and analyze the ChIP-seq data. For custom code that has been deposited into a community repository, provide accession details.*

Flow Cytometry

Plots

Confirm that:

- [x] The axis labels state the marker and fluorochrome used (e.g. CD4-FITC).
- [x] The axis scales are clearly visible. Include numbers along axes only for bottom left plot of group (a 'group' is an analysis of identical markers).
- [ ] All plots are contour plots with outliers or pseudocolor plots.
- [ ] A numerical value for number of cells or percentage (with statistics) is provided.

Methodology

Sample preparation	All cells were extracted immediately after mice were sacrificed and derived from mouse lung tissue. Single cell suspensions were generated as described in the method section. Detailed information on stainings and use of antibodies is also stated in the method section.
Instrument	BD LSRII
Software	Cell Diva, FlowJo
Cell population abundance	purity of single cell samples was manually determined.
Gating strategy	FSC/SSC was used to gate cells in healthy and treated mice. For the analysis of only Epcam+ epithelial cells, a CD45 gate was chosen. Epithelial cells, as well as boundaries between positive and negative populations were defined manually.

- [x] Tick this box to confirm that a figure exemplifying the gating strategy is provided in the Supplementary Information.

Magnetic resonance imaging

Experimental design

Design type	*Indicate task or resting state; event-related or block design.*

Design specifications	*Specify the number of blocks, trials or experimental units per session and/or subject, and specify the length of each trial or block (if trials are blocked) and interval between trials.*
Behavioral performance measures	*State number and/or type of variables recorded (e.g. correct button press, response time) and what statistics were used to establish that the subjects were performing the task as expected (e.g. mean, range, and/or standard deviation across subjects).*

Acquisition

Imaging type(s)	*Specify: functional, structural, diffusion, perfusion.*
Field strength	*Specify in Tesla*
Sequence & imaging parameters	*Specify the pulse sequence type (gradient echo, spin echo, etc.), imaging type (EPI, spiral, etc.), field of view, matrix size, slice thickness, orientation and TE/TR/flip angle.*
Area of acquisition	*State whether a whole brain scan was used OR define the area of acquisition, describing how the region was determined.*

Diffusion MRI ☐ Used ☐ Not used

Preprocessing

Preprocessing software	*Provide detail on software version and revision number and on specific parameters (model/functions, brain extraction, segmentation, smoothing kernel size, etc.).*
Normalization	*If data were normalized/standardized, describe the approach(es): specify linear or non-linear and define image types used for transformation OR indicate that data were not normalized and explain rationale for lack of normalization.*
Normalization template	*Describe the template used for normalization/transformation, specifying subject space or group standardized space (e.g. original Talairach, MNI305, ICBM152) OR indicate that the data were not normalized.*
Noise and artifact removal	*Describe your procedure(s) for artifact and structured noise removal, specifying motion parameters, tissue signals and physiological signals (heart rate, respiration).*
Volume censoring	*Define your software and/or method and criteria for volume censoring, and state the extent of such censoring.*

Statistical modeling & inference

Model type and settings	*Specify type (mass univariate, multivariate, RSA, predictive, etc.) and describe essential details of the model at the first and second levels (e.g. fixed, random or mixed effects; drift or auto-correlation).*
Effect(s) tested	*Define precise effect in terms of the task or stimulus conditions instead of psychological concepts and indicate whether ANOVA or factorial designs were used.*

Specify type of analysis: ☐ Whole brain ☐ ROI-based ☐ Both

Statistic type for inference (See Eklund et al. 2016)	*Specify voxel-wise or cluster-wise and report all relevant parameters for cluster-wise methods.*
Correction	*Describe the type of correction and how it is obtained for multiple comparisons (e.g. FWE, FDR, permutation or Monte Carlo).*

Models & analysis

n/a	Involved in the study
☐	☐ Functional and/or effective connectivity
☐	☐ Graph analysis
☐	☐ Multivariate modeling or predictive analysis

Functional and/or effective connectivity	*Report the measures of dependence used and the model details (e.g. Pearson correlation, partial correlation, mutual information).*
Graph analysis	*Report the dependent variable and connectivity measure, specifying weighted graph or binarized graph, subject- or group-level, and the global and/or node summaries used (e.g. clustering coefficient, efficiency, etc.).*
Multivariate modeling and predictive analysis	*Specify independent variables, features extraction and dimension reduction, model, training and evaluation metrics.*
