# Supplementary material for: Alveolar regeneration through a Krt8+ transitional stem cell state that persists in human lung fibrosis
Source: Nat Commun. 2020 Jul 16;11:3559. doi: 10.1038/s41467-020-17358-3 (PMC7366678; doi:10.1038/s41467-020-17358-3)
Supplement: Supplementary file 3 — Description of Additional Supplementary Files [file 41467_2020_17358_MOESM3_ESM.pdf]

Description of Additional Supplementary Files

File Name: Supplementary Data 1
Description: Cell type marker genes from whole lung dataset

File Name: Supplementary Data 2
Description: Cell type resolved differential gene expression after bleomycin

File Name: Supplementary Data 3
Description: Cell type marker genes from high resolution epithelial cell dataset

File Name: Supplementary Data 4
Description: Cell state trajectory – convergence of airway and alveolar stem cells

File Name: Supplementary Data 5
Description: Cell state trajectory – Krt8+ ADI to AT1

File Name: Supplementary Data 6
Description: Receptor-ligand connectome
